# Lysosome Functions in Atherosclerosis: A Potential Therapeutic Target

**DOI:** 10.3390/cells14030183

**Published:** 2025-01-24

**Authors:** Zhengchao Wang, Xiang Li, Alexandra K. Moura, Jenny Z. Hu, Yun-Ting Wang, Yang Zhang

**Affiliations:** 1 Department of Pharmacological and Pharmaceutical Sciences, College of Pharmacy, University of Houston, Houston, TX 77204, USA; zwang205@central.uh.edu (Z.W.); akmoura@cougarnet.uh.edu (A.K.M.); jzhu31@cougarnet.uh.edu (J.Z.H.); ywang264@central.uh.edu (Y.-T.W.); 2 Provincial Key Laboratory for Developmental Biology and Neurosciences, College of Life Sciences, Fujian Normal University, Fuzhou 350007, China

**Keywords:** lysosomes, autophagy, macrophages, smooth muscle cells, endothelial cells, atherosclerosis

## Abstract

Lysosomes in mammalian cells are recognized as key digestive organelles, containing a variety of hydrolytic enzymes that enable the processing of both endogenous and exogenous substrates. These organelles digest various macromolecules and recycle them through the autophagy–lysosomal system. Recent research has expanded our understanding of lysosomes, identifying them not only as centers of degradation but also as crucial regulators of nutrient sensing, immunity, secretion, and other vital cellular functions. The lysosomal pathway plays a significant role in vascular regulation and is implicated in diseases such as atherosclerosis. During atherosclerotic plaque formation, macrophages initially engulf large quantities of lipoproteins, triggering pathogenic responses that include lysosomal dysfunction, foam cell formation, and subsequent atherosclerosis development. Lysosomal dysfunction, along with the inefficient degradation of apoptotic cells and the accumulation of modified low-density lipoproteins, negatively impacts atherosclerotic lesion progression. Recent studies have highlighted that lysosomal dysfunction contributes critically to atherosclerosis in a cell- and stage-specific manner. In this review, we discuss the mechanisms of lysosomal biogenesis and its regulatory role in atherosclerotic lesions. Based on these lysosomal functions, we propose that targeting lysosomes could offer a novel therapeutic approach for atherosclerosis, shedding light on the connection between lysosomal dysfunction and disease progression while offering new insights into potential anti-atherosclerotic strategies.

## 1. Introduction

In 1974, Christian de Duve was awarded the Nobel Prize for his discovery of an acidic organelle, the lysosome [1,2]. His research, which involved a cholesterol-rich diet, revealed that arterial cells can transform into foam cells due to lysosomal dysfunction, impairing the cells’ ability to process lipoproteins [2]. Lysosomes, often referred to as the digestive organs of cells, are essential for recycling intracellular waste products. Recent studies have expanded our understanding of lysosomes, highlighting their roles not only in degradation but also in secretion, immune responses, and nutrient sensing [3,4]. Lysosomes and their associated signaling pathways are pivotal in various physiological processes and play significant roles in the progression of diseases, including atherosclerosis [5,6].

Atherosclerosis predominantly affects large and medium-sized arteries in inflammatory and metabolic disorders [7,8]. The hallmark of this disease is the formation of atherosclerotic plaques, which result from the excessive deposition of lipids in the arterial walls [9,10]. In the early stages of atherosclerosis, various risk factors induce vascular wall damage, leading to endothelial dysfunction [11]. Mononuclear cells then penetrate the endothelium, accumulating and differentiating into macrophages. Disruptions in arterial lipid metabolism promote the transformation of macrophages into foam cells, exacerbating the inflammatory response and triggering apoptosis [12,13]. As atherosclerosis progresses, VSMCs undergo phenotypic changes, proliferate, and migrate, all of which contribute to plaque formation [14]. Additionally, VSMCs can transform into foam cells by engulfing large quantities of oxLDL in atherosclerotic plaques [15].

During the formation of atherosclerotic plaques, macrophages and VSMCs engulf excessive lipoproteins, triggering a pathogenic response that leads to lysosomal dysfunction. As a result, these cells transform into foam cells, contributing to the development of atherosclerosis [13,16]. Lysosomes are tasked with processing the large quantities of lipoproteins taken up by foam cells and the increasing number of apoptotic cells. In addition, lysosomes serve as nutrient-sensing centers, playing a crucial role in regulating cellular metabolism [13]. Functional defects in lysosomes, which lead to the accumulation of macromolecules, can result in cellular damage [17]. Thus, lysosomal dysfunction, following the excessive uptake of modified LDL, becomes an inevitable feature of atherosclerotic lesions [12,13]. The factors and mechanisms underlying atherosclerosis are complex and require further investigation. Therefore, understanding the intrinsic mechanisms of plaque formation is essential for developing more targeted therapeutic strategies for atherosclerosis in the future.

In this review, we explore lysosome biogenesis, the development of atherosclerotic lesions, and the critical role of lysosomes in atherosclerosis. We also propose new therapeutic strategies targeting lysosomes for the treatment of atherosclerosis. These insights offer a deeper understanding of vascular pathobiology, elucidate the relationship between lysosomal function and atherosclerosis, and pave the way for the development of more effective treatments for vascular diseases.

## 2. Lysosomal Biogenesis and Functions

Lysosomes are membrane-bound organelles composed of an acidic interior enclosed by a phospholipid bilayer. Within the cavity, numerous acidic hydrolases catalyze hydrolysis reactions, breaking down biopolymers such as carbohydrates and lipids [18]. These hydrolases, like other proteins, are synthesized in the ER and undergo modification in the Golgi apparatus. Hydrolases labeled with M6P are then specifically transported to the lysosomes [13,19].

**Figure 1:**
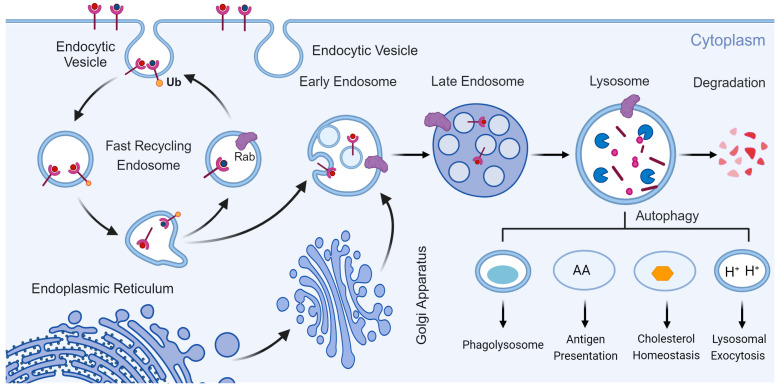
The biosynthesis and main functions of lysosomes. The biosynthesis of lysosomes requires the integration of endocytosis and biosynthetic pathways, and their main function is to degrade and recycle intracellular and extracellular substances, in addition to some other physiological activities. Created with BioRender.com.

Lysosome biosynthesis involves the coordination of endocytosis and biosynthetic pathways. Late endosomes and vesicles derived from the Golgi apparatus fuse to form new lysosomes (Figure 1). Newly synthesized lysosomal hydrolases are directed to lysosomes through two main pathways: direct transport via M6P receptor-mediated mechanisms [19] or indirect transport via the plasma membrane followed by endocytosis [20]. For example, hydrolases are modified by oligosaccharide transferases and GlcNAc-1-phosphate transferases to add M6P residues [21]. At a pH of 6.7, the M6P-labeled proteins bind to M6P receptors in the Golgi complex, and, at pH 6.0, these proteins are released into endosomes [22]. Vesicles containing these hydrolases bud off from the Golgi apparatus, undergo membrane fusion and fission, and ultimately associate with late endosomes, maturing into lysosomes [3,4]. In addition to M6P receptors, two other sorting receptors sortilins and LIMP-2 have been identified for recognizing and directing M6P-labeled proteins [23].

Lysosomal biogenesis is tightly regulated to maintain cellular homeostasis. Current research indicates that their biosynthesis is regulated by the TFEB [24,25], a member of the MiT/TFE family, which contains adjacent basic helix–loop–helix and leucine zipper domains. TFEB was originally cloned from a B lymphocyte cDNA library [26]. Through bioinformatics, Sardiello et al. identified common DNA sequences in the promoters of 96 lysosomal genes, known as CLEAR motifs [27]. TFEB is the key transcription factor that controls lysosomal biosynthesis by positively regulating genes within the CLEAR network [27]. TFEB can be phosphorylated by various kinases, such as mTORC1 [28], ERK [29], MAP4K3 [30], and PKB [31]. The phosphorylation of TFEB inhibits its translocation to the nucleus. The dephosphorylation of TFEB by calmodulin phosphatase calcineurin [32] and PP2A [33] enables its nuclear entry, where it regulates the transcription of target genes, including those in the CLEAR network, which are involved in lysosomal structure and function [34]. The CLEAR motif is a cis-regulatory DNA sequence found in the promoter regions of genes involved in lysosomal biogenesis, autophagy, and other cellular clearance processes [27]. It is characterized by a conserved consensus sequence (GTCACGTGAC) that serves as a binding site for the transcription factor TFEB [27]. The CLEAR network plays a critical role in cellular homeostasis and is tightly regulated by intracellular signaling pathways [34].

The primary function of lysosomes is to degrade and recycle extracellular substances through endocytosis, pinocytosis, and phagocytosis [35]. They also degrade and recover intracellular components via autophagy (Figure 1). Lysosomes eliminate foreign pathogens through phagocytosis, prevent pathogens from entering cells via endocytosis, and utilize hydrolytic enzymes to kill various pathogens in an oxygen-independent manner [36]. In addition to phagocytosis, lysosomes play a critical role in the breakdown of intracellular substrates via autophagy, thus maintaining intracellular homeostasis [24]. This autophagic process involves the formation of autophagy–lysosome complexes [37]. Previous research has shown that molecules like NAADP, ceramide, and cytoplasmic Ca^2+^ levels regulate lysosomal transport and fusion [38,39,40]. Importantly, lysosomes are not only involved in recycling cellular components but also serve as key signaling centers, capable of sensing and integrating changes in the external environment [35]. The metabolic state of the cell is conveyed through the MiT/TFE pathway, which governs lysosomal biogenesis, autophagy, and exocytosis [41]. Lysosomal function requires two types of proteins: soluble lysosomal hydrolases and intact LMP [42]. Moreover, lysosomal exocytosis occurs in a Ca^2+^/SYT7-dependent manner.

Additionally, lysosomes contribute to cell membrane repair through their phospholipid bilayer, which is composed of phosphatidylcholine, phosphatidylglycerol, and sphingomyelin [43]. These phospholipid molecules exhibit amphiphilic properties, enabling the lysosomal membrane to separate the cytoplasm from the lysosomal environment [44]. Lysosomal membranes are enriched with diverse proteins, which can be categorized into two functional groups. The first group comprises proteins involved in enzymatic activity and ion exchange within lysosomes, such as ATPase and H+ transporters [43,44,45,46]. The second group includes proteins associated with cell signaling and the recognition of external molecules, such as receptor proteins and transporters [46]. These proteins enable the lysosomal membrane to mediate material transport and signal transduction.

Decreased lipase activity in premature atherosclerosis exemplifies the close relationship between lysosomal dysfunction and cardiovascular pathogenesis [47]. Zhang et al. demonstrated that lysosomes are pivotal in atherosclerosis, particularly in regulating cell metabolism and the inflammatory response [48]. Skeeni et al. highlighted that cholesterol accumulation in lysosomes is strongly associated with inflammation, thereby promoting the development of atherosclerosis [49]. In summary, lysosomal dysfunction is strongly linked to the development of atherosclerosis.

## 3. The Pathogenesis of Atherosclerosis

**Figure 2:**
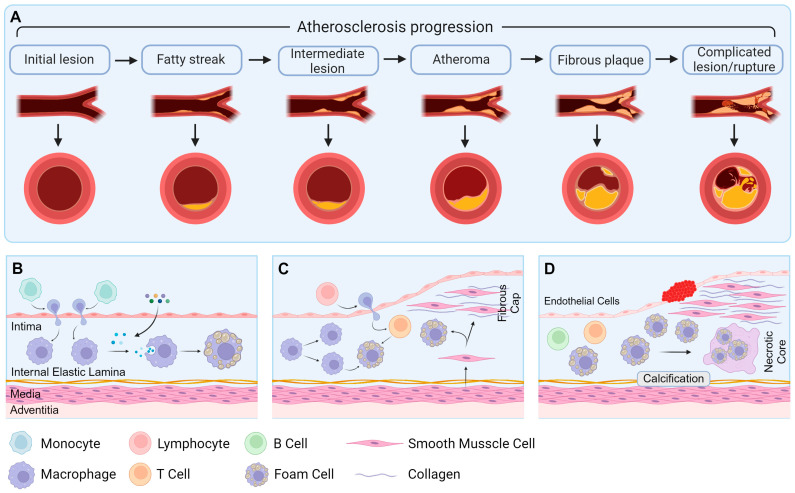
The pathological changes in atherosclerosis. (**A**) The progression of atherosclerosis. (**B**) The fat streak lesion during the early atherosclerosis. (**C**) The pathological change in early atherosclerosis. (**D**) The pathological change in advanced atherosclerosis. Created with BioRender.com.

Atherosclerosis serves as the underlying cause of various cardiovascular pathologies and is a leading contributor to mortality [50]. The onset and progression of atherosclerosis involve the interaction of multiple mechanisms. These mechanisms determine the cytokines and cellular components (Figure 2A), such as ECs, VSMCs, and macrophages, that participate in the process [51,52]. Atherosclerotic lesions are characterized by the accumulation and transformation of lipids, inflammatory cells, VSMCs, and necrotic cell debris in the intimal layer beneath the endothelial monolayer of the vessel lining. These lesions typically progress through three stages: fatty streak development, early atherosclerotic lesion formation, and advanced atherosclerotic lesion progression [52,53].

In the early stages of atherosclerosis (Figure 2B), the vascular wall is subjected to various stimuli that result in endothelial injury and dysfunction [11]. Mononuclear cells then penetrate the damaged endothelium, accumulate in the subendothelial space, and differentiate into macrophages. Due to OS, lipoproteins undergo oxidation, forming oxLDL. Monocytes penetrate the endothelium capture and modify circulating lipoprotein particles, marking the first detectable changes in atherosclerotic lesion development [54]. During plaque formation, macrophages become activated, upregulate scavenger receptors, and begin to uptake modified lipoprotein particles. The phagocytosis of these cholesterol-laden particles leads to the transformation of macrophages into foam cells. The accumulation of foam cells is characteristic of fatty streak lesions, which gradually progress into more advanced fibro-lipidic plaques [55]. Lipid metabolic disorders exacerbate foam cell formation and the inflammatory response [9]. Hence, the interplay between oxidative stress and inflammation is pivotal in the development of atherosclerosis.

During the early stages of atherosclerotic plaque formation (Figure 2C), VSMCs undergo phenotypic transformation, proliferation, and migration in response to vascular injury [14]. Stimulatory signals released by inflammatory cells trigger the translocation of VSMCs from the media to the intima of the arterial wall. Upon migration, VSMCs lose their contractile phenotype and adopt a synthetic phenotype. These migrating VSMCs proliferate and synthesize abnormal ECM proteins, thereby contributing to plaque formation through the establishment of fibrous caps [56]. Dedifferentiated VSMCs also express and produce cytokines that are involved in cell adhesion and inflammation [57], and they serve as a significant source of foam cells in atherosclerotic plaques [15].

During the process of advanced atherosclerosis (Figure 2D), foam cells undergo degeneration, resulting in the formation of a necrotic core composed of cellular debris and cholesterol [6]. Simultaneously, calcification occurs in the intima or media of the artery. Plaque instability and rupture, which are key events in the progression of arterial lesions, are primarily associated with the abnormal activation of MMPs, enzymes that play a critical role in cell migration and ECM degradation [58]. When intravascular lesions rupture or endothelial cells collapse, these events can trigger thrombosis, potentially leading to myocardial infarction or cerebral infarction. In the absence of significant remodeling, atherosclerotic plaques often result in substantial arterial stenosis, restricting blood flow and ultimately causing tissue ischemia [59].

## 4. Lysosome Functions in Vascular Cells of Atherosclerosis

Atherosclerosis involves multifactorial mechanisms and multiple cell types, including immune cells, ECs, and VSMCs (Figure 2). In addition, the autophagy lysosomal pathway plays distinct roles in different cell types during atherosclerosis.

### 4.1. Lysosome Functions in Endothelial Cells

**Figure 3:**
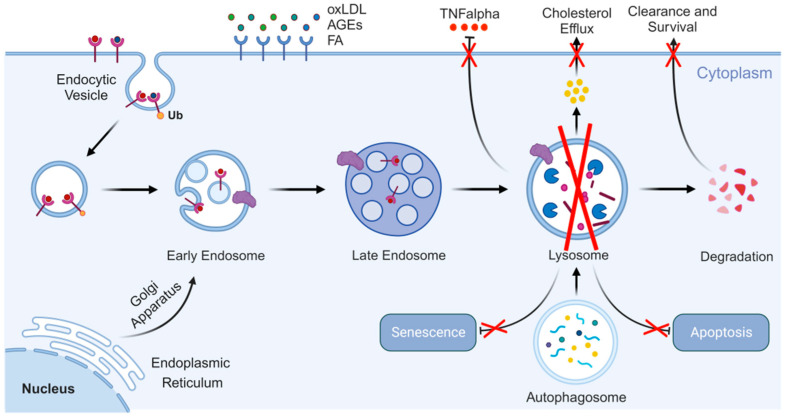
Lysosome functions in endothelial cells of atherosclerosis. Lysosomes are vital to maintain the homeostasis of endothelial cells, and their dysfunction will lead to apoptosis and senescence. Created with BioRender.com.

ECs form a natural barrier to the vasculature, and healthy ECs are essential for vascular structure and function [60], thus ensuring the homeostasis of the arterial intima (Figure 3). Various factors can cause EC damage, triggering the expression of multiple effectors and weakening the endothelial barrier, which serves as an early step in the development of atherosclerosis [61]. During endothelial injury (Figure 3), LDL-C enters the intima, where it accumulates and is subsequently converted into oxLDL through oxidation in the endothelium [62].

In ECs, oxLDL induces autophagy as a protective response [63]. Upon absorption, oxLDL is transported to autophagolysosomes for degradation. This process is triggered by ER stress caused by oxLDL [63]. Other atherosclerotic factors also activate autophagy in ECs, helping prevent endothelial damage [58]. Shear stress resulting from increased blood flow further stimulates autophagy in ECs within the vessel wall [64]. Moreover, palmitic acid induces PINK1-Parkin-mediated mitophagy in ECs, maintaining mitochondrial quality control and preventing endothelial damage [65]. Beyond regulating EC survival, autophagy may play additional roles [66]. Two independent studies have demonstrated that activating endothelial autophagy limits atherosclerotic plaque formation, whereas defects in endothelial autophagy promote plaque development [64].

These findings suggest that autophagy protects ECs from lipid oxidation, metabolic stress, and inflammation in the early stages of atherosclerosis, thereby inhibiting its progression [67].

### 4.2. Lysosome Functions in Smooth Muscle Cells

**Figure 4:**
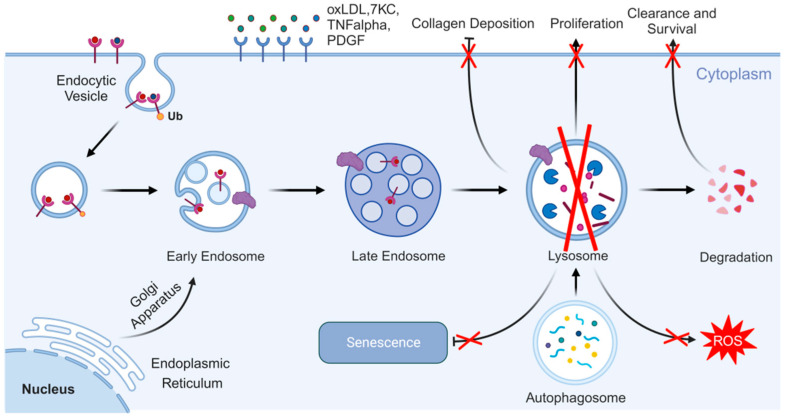
Lysosome functions in vascular smooth muscle cells of atherosclerosis. Lysosomes are vital to maintain the phenotype and function of vascular smooth muscle cells in addition to their survival and differentiation. Created with BioRender.com.

In blood vessels, VSMCs are located in the inner layer of the vessel wall, which play a crucial role in the development of atherosclerosis by promoting neointimal formation [68]. When exposed to chemokines such as CXCL10 and MMPs, VSMCs proliferate abnormally, migrate to the intimal layer of the vessel wall, and undergo a phenotypic transformation into a synthetic phenotype, resulting in a loss of contractile function [69]. In addition to regulating VSMC survival, the autophagy–lysosomal pathway also influences VSMC phenotype and function (Figure 4). Indeed, defects in autophagy in VSMCs can promote their proliferation and migration, thereby contributing to the progression of atherosclerotic plaques [70]. The autophagy–lysosomal pathway is also directly involved in VSMC differentiation. For instance, the P2RY12 receptor inhibits autophagy and promotes the transformation of VSMCs into foam cells by activating the PI3K/Akt/mTOR signaling pathway [71]. This finding parallels observations in macrophages, where reduced autophagic flux impairs lipid clearance during foam cell differentiation [72].

Atherosclerotic plaque formation and ECM deposition are the primary contributors to the pathological thickening of the early atherosclerotic intima. Intimal VSMCs play a critical role in maintaining collagen levels in the fibrous cap and ensuring the stability of atherosclerotic plaques, which helps prevent plaque rupture [69]. Defective autophagy can enhance VSMC cell death and calcification, leading to plaque instability and rupture [73]. As atherosclerosis progresses, the fibroproliferative responses of intimal VSMCs contribute to the healing and repair of arterial injury. However, with prolonged atherogenic stimulation, this repair process becomes dominant, resulting in ECM accumulation, luminal narrowing, reduced blood flow, and ischemia [74].

Additionally, even in the absence of abundant lipids in the blood, VSMCs can still internalize oxLDL which induces apoptosis and results in the release of free cholesterol [75].

### 4.3. Lysosome Functions in Macrophage

Macrophages play a critical role in the development of atherosclerosis. In the early stages of the disease, adhesion factors and chemokines trigger the adhesion and migration of monocytes into the subendothelial layer of the vessel wall [76]. Once there, they differentiate into macrophages in response to M-CSF and GM-CSF stimulation [77]. M1 macrophages secrete pro-inflammatory cytokines [78], while M2 macrophages release anti-atherosclerotic cytokines [79].

Macrophages are not only a primary source of oxidative stress in atherosclerosis, but they can also regulate or be influenced by extracellular oxidative stress [4]. Nox is the main source of oxidative stress in macrophages, and Nox-derived ROS play a critical role in monocyte differentiation [80]. Additionally, mitochondria significantly contribute to oxidative stress in macrophages [81]. Both Nox-derived and mitochondrial ROS are involved in vascular inflammation and the formation of atherosclerotic plaques [82,83]. Macrophage-related inflammation persists throughout the progression of atherosclerosis [84]. Key signaling pathways, including inflammasome, MAPK, PI3K/AKT, TLR, and NF-κB, are heavily involved in this process [53,85]. Recent studies have shown that the autophagy–lysosomal pathway in macrophages can mitigate oxidative stress and inflammation [86].

**Figure 5:**
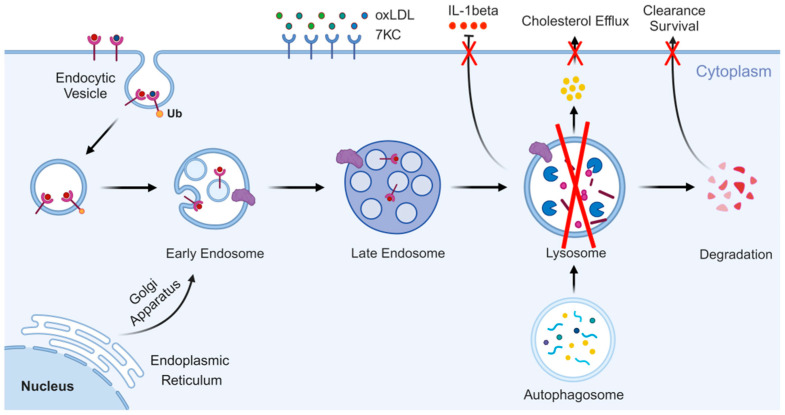
Lysosome functions in macrophages of atherosclerosis. Lysosomes are vital to maintain the functions of macrophages, and their dysfunction will promote the formation of foam cells. Created with BioRender.com.

During the development of atherosclerosis, macrophage scavenger receptors (SRs) recognize and internalize oxLDL through endocytosis, leading to the formation of lipid-rich foam cells (Figure 5). Functional lysosomes in macrophages are essential for the efficient clearance of endocytic substances and preventing atherosclerotic plaque formation. Foam cells, which are a major component of plaques, exhibit impaired autophagy–lysosomal pathways, which are linked to increased oxidative stress and ER stress [87]. Deficiencies in lysosome-dependent endocytic signaling in macrophages inhibit the reverse transport of intracellular cholesterol, leading to cholesterol retention within the cells [88,89]. Under appropriate conditions, macrophages can perform reverse cholesterol transport [58,90,91]. CD36 has been shown to regulate lysosomal Ca^2+^ signaling and the trafficking and fusion of autophagosomes with lysosomes [92].

Additionally, impaired endocytosis can lead to the release of intracellular contents, such as thrombotic factors, that destabilize plaques, stimulate angiogenesis, and ultimately exacerbate atherosclerosis [93].

### 4.4. Lysosome Functions in Stem/Progenitor Cells

**Figure 6:**
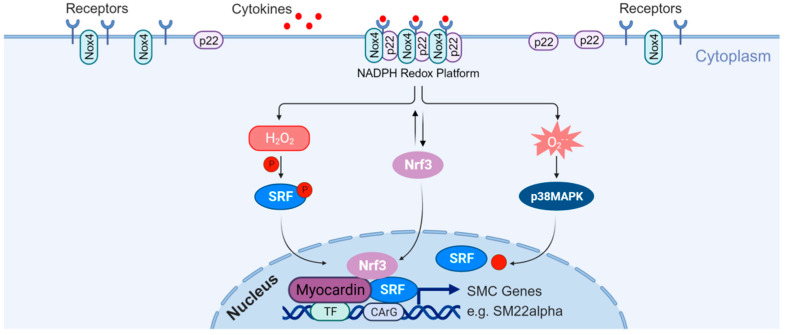
Effects of stem/progenitor cells on atherosclerosis. Stem/progenitor cells are resident in vascular tissues and differentiated as various vascular cells, which could be used to treat atherosclerosis. Created with BioRender.com.

Stem/progenitor cells in vascular tissues possess the ability to differentiate into various vascular cell types [94,95], offering therapeutic potential for atherosclerosis treatment [96]. Oxidative stress plays a critical role in the development of atherosclerosis, and ROS are involved in promoting stem cell differentiation into SMCs. This process is particularly significant for neointimal formation and plaque stability after angioplasty [97,98,99]. Xiao et al. demonstrated that H_2_O_2_, derived from Nox4, promotes the differentiation of stem cells into SMCs, while silencing Nox4 inhibits this differentiation. The prolonged activation of Nox4 enhances SMC differentiation and upregulates SMC markers [97]. Nox4-derived H_2_O_2_ also triggers the phosphorylation and nuclear translocation of SRF [98]. SRF binds to the CArG element and recruits myocardin, forming the SRF/myocardin complex (Figure 6), which regulates Nox4-mediated differentiation [99].

Furthermore, Nrf3 is a crucial factor in regulating SMC differentiation by controlling ROS production. Pepe et al. demonstrated that Nrf3 is essential for the differentiation of stem cells into SMCs [100]. During the early stages of differentiation, Nrf3 in the ER may directly participate in the formation of the SRF/myocardin complex [100]. Simultaneously, cytoplasmic Nrf3 can induce Nox4-mediated ROS production, further triggering differentiation (Figure 6). Therefore, the Nox4/Nrf3-mediated signaling pathway jointly regulates stem cell differentiation into SMCs and influences neointima formation and plaque stability. In addition to oxidative stress, growth factors and cytokines also play a role in regulating SMC differentiation [101,102]. Previous studies have indicated that activation of the autophagy–lysosomal pathway enhances the proliferative capacity of stem cells, and an efficient autophagy–lysosomal pathway is crucial for mitigating stem cell exhaustion and promoting tissue repair [103,104].

The relationship between progenitor/stem cells and lysosomal function in atherosclerosis represents a complex area of study, encompassing diverse biological processes and their intricate interactions. Bautch and Tao et al. demonstrated that progenitor/stem cells are crucial for vascular repair after injury and play a vital role in maintaining arterial homeostasis and functionality. These cells not only contribute to vascular regeneration and repair but also modulate immune-related cellular functions [105]. Bonacina et al. identified immune metabolic reprogramming in atherosclerosis and explored the role of lysosomes in regulating immune responses and stem cell functionality [106]. Seijkens et al. highlighted the therapeutic potential of endothelial progenitor cells, a subpopulation of stem cells, for cardiovascular diseases owing to their self-renewal and differentiation capabilities. These cells are pivotal in vascular regeneration and repair, particularly in the context of atherosclerosis [107].

### 4.5. Lysosome Functions in Lymphocyte Cells

Lymphocytes are intricately involved in the pathogenesis of atherosclerosis [108]. A comprehensive understanding of these cells offers insights into the fundamental mechanisms of atherosclerosis and suggests novel therapeutic approaches. Razeghian-Jahromi et al. demonstrated that macrophages play a dominant role in atherosclerosis, while other immune cells, including T and B lymphocytes, significantly contribute to the regulation of lesions [109].

T lymphocytes recognize and respond to oxLDL, accumulating within atherosclerotic plaques [110,111]. At various stages of atherosclerosis, T cell subsets such as Th1, Th2, and regulatory T cells exert distinct effects on disease progression [112]. Campbell et al. demonstrated that T lymphocytes contribute to both the onset and progression of atherosclerosis [110]. Similarly, Engelen et al. found that T lymphocytes from human atherosclerotic plaques can recognize oxLDL, highlighting their potential involvement in lipid metabolism [111].

Research on the role of B lymphocytes in atherosclerosis remains limited. These cells influence atherosclerosis through antibody production and cytokine secretion [108]. Various B lymphocyte subsets have distinct roles in regulating inflammatory responses and lipid metabolism, which is crucial for understanding the immune mechanisms underlying atherosclerosis [112]. Hedrick et al. identified the role of B lymphocytes in atherosclerosis [108] while Pattarabanjird et al. highlighted B lymphocytes as key regulators of atherosclerosis [112].

**Figure 7:**
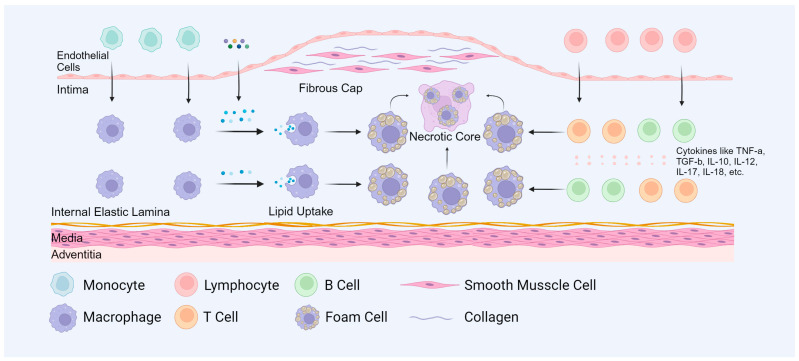
Effects of lymphocyte cells on atherosclerosis. Various lymphocyte cells are involved in the development of atherosclerosis and regulate this process through different cytokines such as TNF-α, TGF-β and interleukins. Created with BioRender.com.

During atherosclerotic lesions, immune cell interactions are pivotal (Figure 7). Lymphocytes interact with other immune cells, including macrophages, to establish a complex immune milieu [109,111]. These interactions influence plaque stability and progression and modulate systemic inflammation, ultimately impacting cardiovascular health [57].

The multiple functions of lymphocytes in atherosclerosis rely on proper lysosomal function, which significantly impacts the disease by regulating immune cell metabolism and inflammatory responses [106]. Vellasamy et al. reported that lymphocyte impairment is influenced not only by intrinsic cellular activity but also by lysosomal integrity. Lysosomal dysfunction can disrupt lymphocyte balance in atherosclerosis pathology [113]. Zhang et al. demonstrated that lysosomal dysfunction partially mediates cytokine secretion in macrophages via the inflammasome, subsequently altering lymphocyte activity and function, and contributing to abnormal immune responses in both atherosclerosis and Gaucher disease [48]. Marques et al. highlighted that lymphocytes rely on intact lysosomal function during atherosclerosis progression, particularly for damaged cell clearance and inflammatory response regulation [114]. Skeyni et al. found that lymphocyte lipid metabolism is intricately linked to lysosomal function, influencing their adaptation to the pathological environment in atherosclerosis [49].

## 5. Impact of Lysosome Functions on the Development of Atherosclerosis

Lysosomes, as lipid-degrading organelles, play a pivotal role in the initiation and progression of atherosclerotic disease. Hence, an in-depth understanding of lysosome-related mechanisms could facilitate the development of novel lysosome-targeted therapies for atherosclerosis.

### 5.1. Endothelial Injury

**Figure 8:**
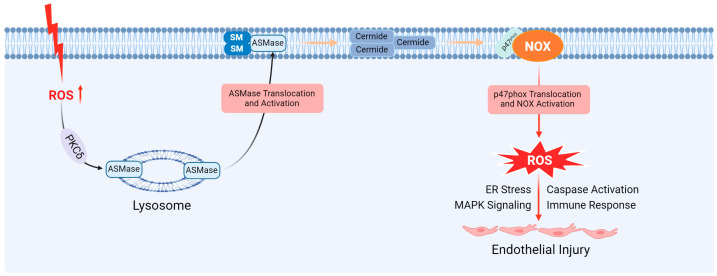
Contribution of ASMase to the LR signalosome and endothelial injury. P47phox is a protein that helps activate the NADPH oxidase enzyme system. Nox: NADPH oxidase. Created with BioRender.com.

Lysosomes regulate EC functions through crosstalk with LR redox signaling in the cell membrane, potentially leading to EC damage under pathological conditions [115]. LRs are sphingolipid- and cholesterol-enriched membrane microdomains that act as signaling platforms to transmit redox signals (Figure 8). Various agonists, such as FasL, promote LR clustering, which facilitates the formation and activation of redox signaling complexes within LR clusters in ECs [116]. The aggregation of Nox subunits is a critical step in activating redox signaling complexes in ECs [115,116]. Many receptors facilitate signaling complex formation by binding to agonists, thereby promoting the development of LR signaling platforms. For instance, polychlorinated biphenyls induce Nox/JAK/EGFR signaling, enhancing immune cell adhesion to the EC layer [117]. In contrast, HDL inhibits Nox by preventing the assembly of Nox subunits in LRs, demonstrating its protective role in the vascular system [118].

Lysosomal dysfunction is harmful and constitutes a hallmark of numerous CVDs [119]. Under pathological stimuli, lysosomes rapidly traffic to and fuse with the cell membrane, triggering localized secretion of ASMase [120]. ASMase hydrolyzes membrane sphingomyelin into ceramide, facilitating LR clustering and the formation of LR redox signaling platforms [121]. The activation of the lysosomal ASMase–ceramide pathway contributes to LR redox signaling induced by agonists, such as FasL-stimulated O_2_·- [122]. Nox-derived O_2_·- plays a role in vascular regulation, but its excessive production damages ECs and promotes atherosclerosis [123]. An increase in lysosomal ASMase activation, driven by ROS, is a pivotal factor in LR-Nox signaling. The formation of ASMase dimers, mediated by modifications to free C-terminal cysteine residues, is essential for enhancing ASMase activity and promoting LR platform formation [27]. The selective activation of lysosomal ASMase enhances lysosomal trafficking and fusion within the LR regions of the endothelial cytoplasmic membrane [124]. Peng et al. indicated that during hypercholesterolemia, the ASMase–ceramide pathway is critical for LR signalosome assembly and activation, contributing to endothelial NLRP3 inflammasome formation, endothelial dysfunction, inflammation, and subsequent atherosclerosis [125].

Additionally, lysosomal membrane destruction is critical in atherosclerotic development, potentially triggered by excessive ROS, leading to lysosomal compartment alterations [43,46].

### 5.2. Inflammasome Activation

**Figure 9:**
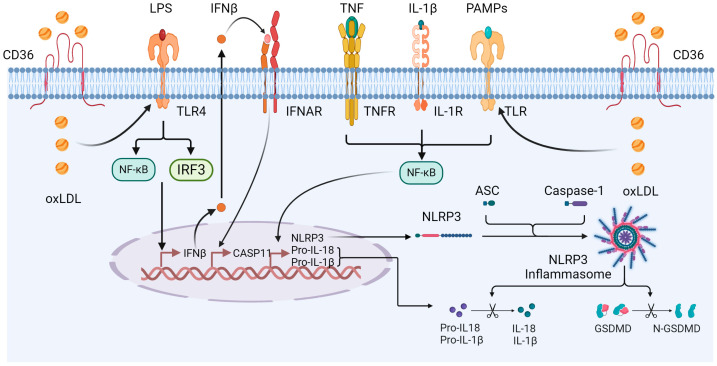
Contribution of CD36 to the activation of NLRP3 inflammasome by oxLDL. PAMPs are molecules that come from microorganisms and are recognized by the immune system. PAMPs: pathogen-associated molecular patterns. Created with BioRender.com.

Atherosclerosis is characterized by the delicate balance between inflammation and regression. The activation of the innate immune system triggers inflammation under pathological conditions and facilitates cardiovascular system remodeling [126]. The NLRP3 inflammasome plays a critical role in the release of mature IL-1β, a key factor in the progression of atherosclerosis (Figure 9). Silencing the NLRP3 inflammasome contributes to the stabilization of atherosclerotic plaques [127].

The NLRP3 inflammasome is predominantly activated in atherosclerosis by oxLDL [128]. Macrophages ingest oxLDL via scavenger receptor CD36, inducing TLR4/TLR6 heterodimer formation and enhancing NF-κB signaling [129,130]. ROS, derived from Nox activity and mitochondrial dysfunction, also activate NLRP3 inflammasomes [131]. Early atherosclerosis is initiated by vascular endothelial injury, where endothelial NO plays a crucial role in maintaining vascular integrity. Subsequently, ROS negate the anti-atherosclerotic and anti-inflammatory effects mediated by NO [132].

The levels of NLRP3, caspase-1, and ASC are significantly elevated in atherosclerotic lesions [133]. The expression of NLRP3 in aortic tissues of atherosclerotic patients correlates with disease severity [134]. ApoE/caspase-1 double knockout models demonstrate a slower progression of atherosclerosis [135]. ASC and caspase-1, as key adaptor proteins of the NLRP3 inflammasome, exhibit sharply increased levels during the progression of atherosclerosis [136]. Although the precise role of the NLRP3 inflammasome in atherosclerotic pathogenesis remains unclear, evidence suggests that NLRP3 regulates IL-1β release through caspase-1 activity, thereby contributing to atherosclerotic progression [137].

### 5.3. Foam Cell Formation

Foam cells play a critical role at all stages of atherosclerosis, from the initial lesion to the advanced plaque formation. Macrophages that accumulate in the intimal layer of the artery are the primary source of foam cells, with a smaller contribution from ECs and VSMCs [138]. The excessive uptake of oxLDL triggers the transformation of vascular cells into foam cells [139].

The initial lesion in atherosclerosis is typically caused by localized increases in lipoproteins within the arterial intima. LDL can penetrate the endothelium or adhere to ECM components, beginning to accumulate in the arterial intima [6], thus promoting the formation of fatty streaks [140]. First, lipoproteins are captured at the lesion site. LDL-C cannot penetrate the endothelial junctions directly but instead enters ECs via endocytosis, leading to an increase in LDL concentration within the intima [140]. Second, the activation of ECs occurs. Oxidized lipids play a critical role in activating ECs, facilitating the migration of leukocytes across the arterial intima [141]. Adhesion and uptake molecules generated by LDL oxidation also play an important role [142]. Monocyte-differentiated macrophages facilitate the uptake of oxidized lipids, such as oxLDL [143]. The third stage involves the activation of leukocytes. At the early stages of atherosclerosis, monocytes and T lymphocytes traverse the endothelial barrier under the influence of chemokines and adhesion molecules. Chemokines are small proteins that play a crucial role in leukocyte activation and migration [144]. Macrophages express large amounts of the chemokine MCP-1 at this stage [145]. Finally, foam cell formation occurs. Mononuclear phagocytes enter the intima, where they differentiate into macrophages. These macrophages absorb and accumulate oxLDL via their scavenger receptors, subsequently transforming into foam cells [146]. As these yellow cells accumulate in large numbers, lipid streaks appear [147].

Additionally, cholesterol esters are crucial in foam cell formation. ACAT1 is an enzyme that converts free cholesterol into cholesterol esters [148], whereas NCEH hydrolyzes cholesterol esters to release free cholesterol [149]. Free cholesterol can be transported out of cells via membrane cholesterol transport systems or passive membrane diffusion [150]. Thus, cholesterol homeostasis, involving both etherification and de-etherification, is crucial during this transformation.

### 5.4. Plaque Development

During the development of atherosclerotic plaques, lysosomes are under significant strain as they must handle the large quantities of lipoproteins absorbed by foam cells and the increasing number of apoptotic bodies engulfed by phagocytes. Therefore, understanding the molecular mechanisms underlying arterial plaque formation is crucial for developing more accurate and effective treatments for atherosclerosis in the future.

Atherosclerotic lesions predominantly occur in the artery intima, particularly in areas of branching and high curvature [9,151], where the endothelium is more permeable and LDL tends to accumulate [152]. In response to atherogenic stimuli, VSMCs begin to secrete large quantities of modified ECM components. This process serves as the foundation for diffuse intimal thickening, commonly referred to as “fat streaks” [153].

The ECM further promotes lipid accumulation in the vascular intima, leading to pathological intimal thickening during the early stages of atherosclerosis LDL, which undergoes modification into pro-inflammatory oxLDL through mechanisms such as oxidation and enzyme cleavage. These modified lipoproteins not only damage the endothelium and increase its permeability but also activate ECs, triggering a pro-inflammatory cascade. Activated ECs secrete chemical attractants and adhesion molecules, such as MCP-1 and ICAM-1 [154], which recruit leukocytes to the vascular wall. Within the intima, monocytes predominantly differentiate into M1 macrophages and phagocytose-modified lipoproteins via SR [155].

Macrophage infiltration and proliferation are characteristic features of pathological intimal thickening, which develops into fibrous atherosclerotic plaques. Macrophages recruit T and B lymphocytes by secreting pro-inflammatory cytokines [156]. VSMCs can differentiate into macrophage-like cells and uptake modified oxLDL [157]. The excessive uptake of oxLDL ultimately damages the lysosomes responsible for degrading lipoproteins. The saturation of lysosomal degradation capacity leads to the accumulation of lipid droplets, promoting the transformation of foam cells [158]. The apoptosis of these foam cells can result in the formation of lipid-rich necrotic cores within fibrous atherosclerotic plaques [159].

Necrotic cores are protected by VSMCs through the formation of a fibrous cap [160], but cytokines produced during inflammation can induce VSMC apoptosis or differentiation, promoting mineral deposition [161]. Ultimately, VSMC death, collagen degradation, and fibrous cap invasion compromise plaque stability, leading to plaque rupture and thrombosis.

### 5.5. Endocytosis and Exocytosis

The lysosomal membrane not only participates in phagocytosis and digestion but also plays a role in cell secretion and the clearance of intracellular waste [162,163]. When cells engulf and digest foreign substances, the lysosomal membrane fuses with the cell membrane, forming phagosomes [162]. These phagosomes then merge with lysosomes to create digestive vesicles containing digestive enzymes, thereby facilitating the digestion of foreign substances [163].

Endocytosis is a crucial process by which cells acquire large molecules from the extracellular environment [164]. In this process, extracellular molecules are enveloped and invaginated by the plasma membrane to form vesicles [165], which then detach and are internalized to participate in various physiological processes. In the early stages of atherosclerosis, monocyte-derived macrophages exhibit rapid and efficient endocytosis of apoptotic cells, which helps limit plaque progression [166]. Disruption of autophagy can impair macrophage clearance of apoptotic cells, promoting plaque necrosis [167]. Damaged lysosomal acidification and reduced hydrolytic enzyme activity affect the macrophage’s ability to process phagocytosed materials [168]. In advanced stages of atherosclerosis, defects in phagocytic clearance exacerbate secondary necrosis, ultimately leading to plaque rupture [169,170].

Exocytosis is the reverse of endocytosis, a process in which substances surrounded by a membrane within the cell are packaged into vesicles that gradually move to the cell surface [162,171]. The vesicle membrane fuses with the plasma membrane, opening outward to release its contents; this process is called exocytosis [162]. Although lysosomes are not typically secretory organelles, they can still release their contents via an unconventional pathway known as lysosomal exocytosis [171]. In this process, the lysosomal contents are secreted after fusion, a crucial step for cellular clearance and maintaining cell health [172]. However, increased lysosomal exocytosis can result in the release of undigested substances into the extracellular space, which may then be engulfed by macrophages, thereby exacerbating atherosclerosis [173]. In atherosclerotic plaques, the levels of various hydrolases are significantly elevated including LAL, cathepsin B, and cathepsin D [174]. For instance, elevated LAL and cathepsin D levels contribute to LDL modification [175]. Additionally, extracellular cathepsin B can degrade the ECM, further increasing plaque vulnerability [176].

### 5.6. Autophagy–Lysosomal Biogenesis

The autophagy–lysosome system is crucial in cardiovascular cells [45]. In atherosclerosis, autophagy can serve as a protective mechanism, while it can also have detrimental effects [177]. For example, damaged mitochondria may be engulfed, preventing the release of pro-apoptotic factors and interfering with apoptosis [178].

**Figure 10:**
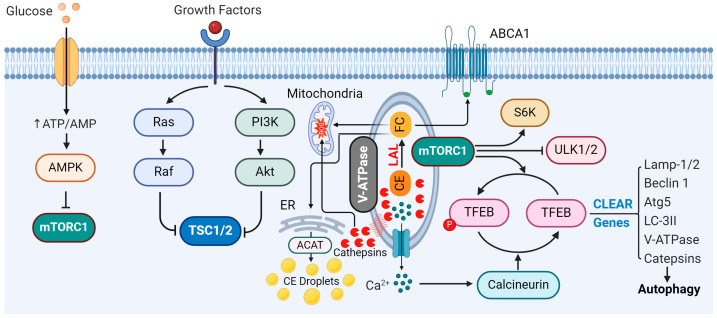
Contribution and regulation of TFEB during autophagy–lysosomal biogenesis. The enzyme acyl-CoA:cholesterol acyltransferase (ACAT) is normally localized in the endoplasmic reticulum (ER). CE: cholesteryl ester, FC: free cholesterol. Created with BioRender.com.

Many studies have demonstrated that autophagy can have both positive and negative effects in a cell-specific and stage-specific manner during atherosclerotic development (Figure 10). For instance, macrophage autophagy not only facilitates the degradation of exogenous and endogenous atherogenic substances in plaques but also reduces macrophage apoptosis and inflammatory IL-1β levels, thereby mitigating atherosclerotic damage [178]. The specific knockout of the autophagy protein ATG5 in macrophages leads to dysfunctional autophagy and exacerbates atherosclerosis [86,167,179]. Endothelial autophagy is critical for lipid homeostasis, but its over-activation can lead to EC damage, thereby enhancing atherosclerotic progression [180]. In SMCs, autophagy can promote SMC differentiation and quiescence, reduce proliferation, and prevent fibrosis. However, excessive autophagy may lead to cell death and increase the instability of atherosclerotic plaques [181]. Autophagy is active in various cardiovascular cells, which helps degrade cellular components through lysosomal pathways, recovers essential catabolites, and ensures cell quality and energy balance [182]. All of these functions are crucial for maintaining vascular system stability, coping with lipid challenges, and preventing atherosclerosis [183].

The cooperation between autophagosomes and lysosomes is essential for the processes of autophagy and catabolism, as their coordinated interaction prevents the accumulation of excessive cargo-filled autophagosomes, thereby maintaining the degradation capacity of lysosomes [45,46]. Furthermore, a mismatch in the quantity of these organelles can have detrimental consequences for cells. Numerous studies have shown that trehalose is an effective autophagy inducer, which not only stimulates autophagy and lysosomal biosynthesis but also provides protection against atherosclerosis [184,185,186].

## 6. Therapeutic Potential of Lysosome in Atherosclerosis

**Figure 11:**
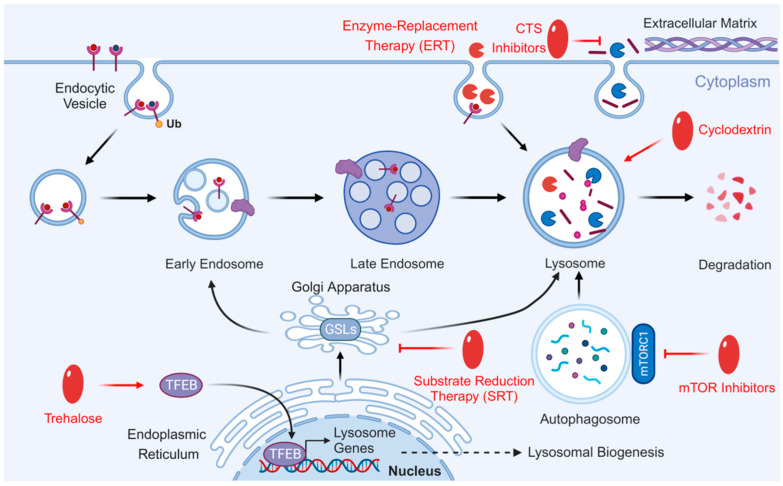
Atherosclerosis treatment targeting lysosomal dysfunction. Various methods and strategies developed by targeting lysosomes could be used to treat atherosclerosis, including ERT, SRT, cathepsin, trehalose, cyclodextrins, and mTOR inhibitors. Created with BioRender.com.

Lysosomal dysfunction is closely associated with the pathological progression of atherosclerosis. Marques et al. reported that declining lysosomal function accelerates atherosclerosis progression [114]. Emanuel et al. suggested that restoring lysosomal function or promoting its biogenesis may lead to novel therapeutic strategies for atherosclerosis [187]. Various methods and strategies developed in the field of LSDs can be employed to target lysosomes for treating atherosclerosis (Figure 11). These methods can directly correct protein defects, mitigate side effects, and enhance lysosomal function, which is highly significant for the ongoing improvement of atherosclerotic therapy.

Enzyme replacement therapy (ERT) continues to be the standard treatment for most LSDs (Figure 11). Lysosomal enzyme synthesis, similar to other proteins, occurs through the ER–Golgi complex; however, some exogenous enzymes are still absorbed and transported to the lysosome, making ERT a viable therapeutic option. The inhibition of LAL in macrophages reduces cholesterol efflux via ABCA1, impairing oxidative sterol production and the phagocytosis of dead cells [188]. Enhanced LAL activity can reduce atherosclerosis in LDL receptor knockout mice [189]. Considering the role of LAL in atherosclerosis, supplementing recombinant LAL enzymes may represent an effective strategy to prevent disease progression [190]. However, the ERT strategy still faces significant limitations regarding the cost and delivery efficiency of recombinant enzymes [191].

Substrate reduction therapy (SRT) is a widely used alternative treatment for LSDs when ERT is contraindicated. SRT involves the inhibition of GSL synthesis, a primary or secondary storage product of LSD (Figure 11). Similar to LSD, GSL accumulates in atherosclerotic lesions in both humans and mice due to impaired lysosomal degradation and is associated with inflammation and plaque instability [192]. Drugs that inhibit glucosylceramide synthase and block GSL synthesis can improve atherosclerosis in mouse models, but further confirmation is required to establish GSL as a therapeutic target for atherosclerosis [193].

Lysosomal cathepsins play a crucial role in maintaining cell homeostasis (Figure 11). Their lysosomal activity in vitro mediates various atherosclerotic processes, such as oxLDL degradation and ECM remodeling [174,194]. Furthermore, their ablation impairs the formation of atherosclerotic plaques [195,196,197]. Therefore, cathepsins may represent a novel therapeutic target for treating atherosclerosis. However, due to their non-specific inhibitory effects, the side effects and efficacy of cathepsin inhibitors require further investigation.

mTOR inhibitors have been extensively studied and shown to regulate the activity of the autophagy–lysosomal system (Figure 11). These inhibitors have demonstrated anti-atherosclerotic effects in numerous studies by promoting plaque clearance and inhibiting inflammation [198,199]. However, a major disadvantage of this approach is dyslipidemia.

Trehalose is not only an effective autophagy inducer but also promotes lysosomal biogenesis [200,201,202], providing protection against atherosclerosis (Figure 11). TFEB is the primary regulatory factor for autophagy and lysosomal biosynthesis, and its overexpression increases lysosome numbers while enhancing their degradative capacity [203,204]. In vitro studies demonstrate that the overexpression of TFEB induces lysosomal biogenesis, rescues lysosomal function, inhibits inflammasome activation, and reduces atherosclerotic progression [187]. In vivo studies further confirm that macrophage overexpression of TFEB reduces atherosclerosis in mouse models [205]. These findings suggest that enhancing the autophagy–lysosome system in macrophages may improve atherosclerosis [187,203,204].

Furthermore, cyclodextrins can facilitate the release of cholesterol from late endosomes and lysosomes into the cytoplasm (Figure 11). Cyclodextrins modulate the production of oxysterols by macrophages, promote LXR-mediated cholesterol efflux, and contribute to the regression of atherosclerosis in ApoE^−/−^ mice [206]. Cyclodextrins not only reduce the cholesterol content in VSMCs and ECs but also influence the expression of ABC transporters [207]. Although cyclodextrins show potential therapeutic effects on atherosclerosis, they are known to cause cyototoxicity and should be used with caution.

In summary, although numerous methods and strategies targeting lysosomes have been developed and show promising therapeutic effects in preclinical studies, many of these compounds still demonstrate limited lysosomal targeting in clinical trials.

## 7. Summary and Prospects

With advancements in lysosomal research, growing evidence suggests that lysosomal dysfunction is pivotal in atherosclerotic development. During vascular disease progression, elevated lipid concentrations generate free radicals, which target the arterial endothelial wall. This activates the endothelium, increases vascular permeability, and initiates the recruitment of inflammatory cells. Monocytes migrating into the intima differentiate into macrophages. These macrophages ingest large amounts of oxLDL, becoming foam cells that form fatty streaks and contribute to the progression of atherosclerosis and atherosclerotic plaque formation. In the early stages of atherosclerotic plaque formation, lysosomal catalytic function in vascular cells remains intact, processing captured lipoproteins effectively. However, excessive substrate intake eventually disrupts lysosomal function.

Furthermore, due to the complexity of atherosclerosis, effective therapeutic drugs have remained scarce for decades. Lysosomal dysfunction could represent a novel target for future therapeutic drug development, given its impact on atherosclerotic pathogenesis. Although significant progress has been made in understanding lysosomal dysfunction in arterial plaque cells, several key issues remain to be addressed, such as lipoprotein modification and the role of oxLDL components. Notably, research on LSDs offers valuable insights and potential research directions. For instance, lysosomal biogenesis, a critical factor in atherosclerotic development, is regulated by TFEB. Additionally, several biomarkers, such as p62 aggregates, extracellular LAL levels, and circulating cathepsin levels, can be utilized for atherosclerotic prognosis. Finally, interventions that address individual lysosomal protein defects or enhance the overall autophagy–lysosomal mechanism hold significant potential for treating atherosclerosis.

In this review, we have summarized the role of lysosomal dysfunction in atherosclerotic formation, which will enhance our understanding of lysosome-related diseases and their mechanisms within the cardiovascular system. Furthermore, building on LSD research, various methods and strategies targeting lysosomes for treating atherosclerosis will emerge as key research directions in the future, carrying significant implications.

## Data Availability

Not applicable.

## Abbreviations

ACAT1: acetyl CoA acetyltransferase; ASC, apoptosis-associated speck-like protein containing a CARD; ASMase, acid sphingomyelinase; CLEAR, coordination of lysosomal expression and regulatory motif; CVD, cardiovascular diseases; EC, endothelial cell; ECM, extracellular matrix; ER, endoplasmic reticulum; ERK, extracellular signal-regulated kinase; GlcNAc, N-acetyl-D-glucosamine; GM-CSF, granulocyte macrophage colony promoting factor; GSL, glycosphingolipid; HDL, high-density lipoprotein; LAL, lysosomal acid lipase. LDL, low-density lipoprotein; LDL-C, low-density lipoprotein cholesterol; LIMP-2, lysosomal integrated membrane protein 2; LMP, lysosomal membrane protein; LR, lipid raft; LSD, lysosomal storage disease; M-CSF, macrophage colony stimulating factor; M6P, mannose-6-phosphate; MAPK, mitogen-activated protein kinase; MCP-1, monocyte chemokine protein; MiT/TFE, the small eye/transcription factor E; MITF, melanocyte inducible transcription factor; MMP, matrix metalloproteinase; mTOR, mammalian target of rapamycin; mTORC1, mammalian target of rapamycin complex 1; NAADP, nicotinate adenine dinucleotide phosphate; NCEH, neutral cholesterol ester hydrolase; NF-κB, nuclear factors κB; NO, nitric oxide; Nox, NADPH oxidase; OS, oxidative stress; oxLDL, oxidized low-density lipoprotein; PI3K, phosphoinositide3 kinase; PKB/Akt, protein kinase B; PP2A, protein phosphatase 2; ROS, reactive oxygen species; SMA, smooth muscle actin; SR, scavenger receptor; SRF, serum response factor; SYT7, synaptic binding protein 7; TFEB, transcription factor EB; TFEC, transcription factor EC; TFE3, transcription factor binding to IGHM enhancer 3; TLR, toll-like receptor; TNF-α, tumor necrosis factor-α; VSMC, vascular smooth muscle cell.

## References

[1] Peters T.J., De Duve C. (1974). Lysosomes of the arterial wall. II. Subcellular fractionation of aortic cells from rabbits with experimantal atheroma. Exp. Mol. Pathol..

[2] Shio H., Farquhar M.G., de Duve C. (1974). Lysosomes of the arterial wall. IV. Cytochemical localization of acid phosphatase and catalase in smooth muscle cells and foam cells from rabbit atheromatous aorta. Am. J. Pathol..

[3] Settembre C., Perera R.M. (2024). Lysosomes as coordinators of cellular catabolism, metabolic signalling and organ physiology. Nat. Rev. Mol. Cell Biol..

[4] Nixon R.A., Rubinsztein D.C. (2024). Mechanisms of autophagy-lysosome dysfunction in neurodegenerative diseases. Nat. Rev. Mol. Cell Biol..

[5] Bonam S.R., Wang F., Muller S. (2019). Lysosomes as a therapeutic target. Nat. Rev. Drug Discov..

[6] Wojtasińska A., Frąk W., Lisińska W., Sapeda N., Młynarska E., Rysz J., Franczyk B. (2023). Novel insights into the molecular mechanisms of atherosclerosis. Int. J. Mol. Sci..

[7] Gencer S., Evans B.R., van der Vorst E.P.C., Döring Y., Weber C. (2021). Inflammatory chemokines in atherosclerosis. Cells.

[8] Aronova A., Tosato F., Naser N., Asare Y. (2023). Innate immune pathways in atherosclerosis-from signaling to long-term epigenetic reprogramming. Cells.

[9] Lusis A.J. (2000). Atherosclerosis. Nature.

[10] Hansson G.K. (2005). Inflammation, atherosclerosis, and coronary artery disease. N. Engl. J. Med..

[11] Jebari-Benslaiman S., Galicia-García U., Larrea-Sebal A., Olaetxea J.R., Alloza I., Vandenbroeck K., Benito-Vicente A., Martín C. (2022). Pathophysiology of atherosclerosis. Int. J. Mol. Sci..

[12] Adkar S.S., Leeper N.J. (2024). Efferocytosis in atherosclerosis. Nat. Rev. Cardiol..

[13] De Meyer G.R.Y., Zurek M., Puylaert P., Martinet W. (2024). Programmed death of macrophages in atherosclerosis: Mechanisms and therapeutic targets. Nat. Rev. Cardiol..

[14] Miano J.M., Fisher E.A., Majesky M.W. (2021). Fate and state of vascular smooth muscle cells in atherosclerosis. Circulation.

[15] Basatemur G.L., Jørgensen H.F., Clarke M.C.H., Bennett M.R., Mallat Z. (2019). Vascular smooth muscle cells in atherosclerosis. Nat. Rev. Cardiol..

[16] Bhat O.M., Li P.L. (2021). Lysosome function in cardiovascular diseases. Cell Physiol. Biochem..

[17] Poznyak A.V., Sukhorukov V.N., Popov M.A., Chegodaev Y.S., Postnov A.Y., Orekhov A.N. (2023). Mechanisms of the Wnt pathways as a potential target pathway in atherosclerosis. J. Lipid Atheroscler..

[18] Ballabio A., Bonifacino J.S. (2020). Lysosomes as dynamic regulators of cell and organismal homeostasis. Nat. Rev. Mol. Cell Biol..

[19] Griffiths G., Hoflack B., Simons K., Mellman I., Kornfeld S. (1988). The mannose 6-phosphate receptor and the biogenesis of lysosomes. Cell.

[20] Ruivo R., Anne C., Sagné C., Gasnier B. (2009). Molecular and cellular basis of lysosomal transmembrane protein dysfunction. Biochim. Biophys. Acta..

[21] Do H., Lee W.S., Ghosh P., Hollowell T., Canfield W., Kornfeld S. (2002). Human mannose 6-phosphate-uncovering enzyme is synthesized as a proenzyme that is activated by the endoprotease furin. J. Biol. Chem..

[22] Bajaj L., Lotfi P., Pal R., Ronza A.D., Sharma J., Sardiello M. (2019). Lysosome biogenesis in health and disease. J. Neurochem..

[23] Reczek D., Schwake M., Schröder J., Hughes H., Blanz J., Jin X., Brondyk W., Van Patten S., Edmunds T., Saftig P. (2007). LIMP-2 is a receptor for lysosomal mannose-6-phosphate-independent targeting of beta-glucocerebrosidase. Cell.

[24] Settembre C., Fraldi A., Medina D.L., Ballabio A. (2013). Signals from the lysosome: A control centre for cellular clearance and energy metabolism. Nat. Rev. Mol. Cell Biol..

[25] Jin Y., Liu Y., Xu L., Xu J., Xiong Y., Peng Y., Ding K., Zheng S., Yang N., Zhang Z. (2022). Novel role for caspase 1 inhibitor VX765 in suppressing NLRP3 inflammasome assembly and atherosclerosis via promoting mitophagy and efferocytosis. Cell Death. Dis..

[26] Peng Z., Zhan H., Shao Y., Xiong Y., Zeng L., Zhang C., Liu Z., Huang Z., Su H., Yang Z. (2020). 13-Methylberberine improves endothelial dysfunction by inhibiting NLRP3 inflammasome activation via autophagy induction in human umbilical vein endothelial cells. Chin. Med..

[27] Ito Y., Maejima Y., Nakagama S., Shiheido-Watanabe Y., Tamura N., Sasano T. (2021). Rivaroxaban, a direct oral factor Xa inhibitor, attenuates atherosclerosis by alleviating factor Xa-PAR2-mediated autophagy suppression. JACC Basic. Transl. Sci..

[28] Li Y., Xu M., Ding X., Yan C., Song Z., Chen L., Huang X., Wang X., Jian Y., Tang G. (2016). Protein kinase C controls lysosome biogenesis independently of mTORC1. Nat. Cell Biol..

[29] Settembre C., Di Malta C., Polito V.A., Garcia Arencibia M., Vetrini F., Erdin S., Erdin S.U., Huynh T., Medina D., Colella P. (2011). TFEB links autophagy to lysosomal biogenesis. Science.

[30] Hsu C.L., Lee E.X., Gordon K.L., Paz E.A., Shen W.C., Ohnishi K., Meisenhelder J., Hunter T., La Spada A.R. (2018). MAP4K3 mediates amino acid-dependent regulation of autophagy via phosphorylation of TFEB. Nat. Commun..

[31] Palmieri M., Pal R., Nelvagal H.R., Lotfi P., Stinnett G.R., Seymour M.L., Chaudhury A., Bajaj L., Bondar V.V., Bremner L. (2017). mTORC1-independent TFEB activation via Akt inhibition promotes cellular clearance in neurodegenerative storage diseases. Nat. Commun..

[32] Medina D.L., Di Paola S., Peluso I., Armani A., De Stefani D., Venditti R., Montefusco S., Scotto-Rosato A., Prezioso C., Forrester A. (2015). Lysosomal calcium signalling regulates autophagy through calcineurin and TFEB. Nat. Cell Biol..

[33] Chen L., Wang K., Long A., Jia L., Zhang Y., Deng H., Li Y., Han J., Wang Y. (2017). Fasting-induced hormonal regulation of lysosomal function. Cell Res..

[34] Palmieri M., Impey S., Kang H., di Ronza A., Pelz C., Sardiello M., Ballabio A. (2011). Characterization of the CLEAR network reveals an integrated control of cellular clearance pathways. Hum. Mol. Genet..

[35] Saftig P., Puertollano R. (2021). How lysosomes sense, integrate, and cope with stress. Trends Biochem. Sci..

[36] Lawrence R.E., Zoncu R. (2019). The lysosome as a cellular centre for signalling, metabolism and quality control. Nat. Cell Biol..

[37] Reggiori F., Klionsky D.J. (2002). Autophagy in the eukaryotic cell. Eukaryot. Cell.

[38] Xu M., Zhang Y., Xia M., Li X.X., Ritter J.K., Zhang F., Li P.L. (2012). NAD(P)H oxidase-dependent intracellular and extracellular O_2_^−^ production in coronary arterial myocytes from CD38 knockout mice. Free Radic. Biol. Med..

[39] Li X., Zhang Y., Xia M., Gulbins E., Boini K.M., Li P.L. (2014). Activation of Nlrp3 inflammasomes enhances macrophage lipid-deposition and migration: Implication of a novel role of inflammasome in atherogenesis. PLoS ONE.

[40] Wang L., Chen Y., Li X., Zhang Y., Gulbins E., Zhang Y. (2016). Enhancement of endothelial permeability by free fatty acid through lysosomal cathepsin B-mediated Nlrp3 inflammasome activation. Oncotarget.

[41] Marques A.R.A., Di Spiezio A., Thießen N., Schmidt L., Grötzinger J., Lüllmann-Rauch R., Damme M., Storck S.E., Pietrzik C.U., Fogh J. (2020). Enzyme replacement therapy with recombinant pro-CTSD (cathepsin D) corrects defective proteolysis and autophagy in neuronal ceroid lipofuscinosis. Autophagy.

[42] Eskelinen E.L., Tanaka Y., Saftig P. (2003). At the acidic edge: Emerging functions for lysosomal membrane proteins. Trends Cell Biol..

[43] Reynolds T. (2013). Cholesteryl ester storage disease: A rare and possibly treatable cause of premature vascular disease and cirrhosis. J. Clin. Pathol..

[44] Zhang Z., Yue P., Lu T., Wang Y., Wei Y., Wei X. (2021). Role of lysosomes in physiological activities, diseases, and therapy. J. Hematol. Oncol..

[45] Skeyni A., Pradignac A., Matz R.L., Terrand J., Boucher P. (2024). Cholesterol trafficking, lysosomal function, and atherosclerosis. Am. J. Physiol. Cell Physiol..

[46] Nayor M., Brown K.J., Vasan R.S. (2021). The molecular basis of predicting atherosclerotic cardiovascular disease risk. Circ. Res..

[47] Stroope C., Nettersheim F.S., Coon B., Finney A.C., Schwartz M.A., Ley K., Rom O., Yurdagul A. (2024). Dysregulated cellular metabolism in atherosclerosis: Mediators and therapeutic opportunities. Nat. Metab..

[48] Döring Y., van der Vorst E.P.C., Weber C. (2024). Targeting immune cell recruitment in atherosclerosis. Nat. Rev. Cardiol..

[49] Björkegren J.L.M., Lusis A.J. (2022). Atherosclerosis: Recent developments. Cell.

[50] Skålén K., Gustafsson M., Rydberg E.K., Hultén L.M., Wiklund O., Innerarity T.L., Borén J. (2002). Subendothelial retention of atherogenic lipoproteins in early atherosclerosis. Nature.

[51] Hou P., Fang J., Liu Z., Shi Y., Agostini M., Bernassola F., Bove P., Candi E., Rovella V., Sica G. (2023). Macrophage polarization and metabolism in atherosclerosis. Cell Death Dis..

[52] Allahverdian S., Chaabane C., Boukais K., Francis G.A., Bochaton-Piallat M.L. (2018). Smooth muscle cell fate and plasticity in atherosclerosis. Cardiovasc. Res..

[53] Libby P. (2002). Inflammation in atherosclerosis. Nature.

[54] Glass C.K., Witztum J.L. (2001). Atherosclerosis. the road ahead. Cell.

[55] Glagov S., Weisenberg E., Zarins C.K., Stankunavicius R., Kolettis G.J. (1987). Compensatory enlargement of human atherosclerotic coronary arteries. N. Engl. J. Med..

[56] He L., Zhang C.L., Chen Q., Wang L., Huang Y. (2022). Endothelial shear stress signal transduction and atherogenesis: From mechanisms to therapeutics. Pharmacol. Ther..

[57] Zhang F., Li J., Gu C., Zhang H. (2022). MiR-140-5p upregulation suppressed beta-glycerophosphate-induced vascular smooth muscle cell calcification via targeting TLR4. Immunopharmacol. Immunotoxicol..

[58] Chen Q., Qi X., Zhang W., Zhang Y., Bi Y., Meng Q., Bian H., Li Y. (2021). Catalpol inhibits macrophage polarization and prevents postmenopausal atherosclerosis through regulating estrogen eeceptor alpha. Front. Pharmacol..

[59] Zhang Y.L., Cao Y.J., Zhang X., Liu H.H., Tong T., Xiao G.D., Yang Y.P., Liu C.F. (2010). The autophagy-lysosome pathway: A novel mechanism involved in the processing of oxidized LDL in human vascular endothelial cells. Biochem. Biophys. Res. Commun..

[60] Vion A.C., Kheloufi M., Hammoutene A., Poisson J., Lasselin J., Devue C., Pic I., Dupont N., Busse J., Stark K. (2017). Autophagy is required for endothelial cell alignment and atheroprotection under physiological blood flow. Proc. Natl. Acad. Sci. USA.

[61] Wu W., Xu H., Wang Z., Mao Y., Yuan L., Luo W., Cui Z., Cui T., Wang X.L., Shen Y.H. (2015). PINK1-Parkin-mediated mitophagy protects mitochondrial integrity and prevents metabolic stress-induced endothelial injury. PLoS ONE.

[62] Torisu T., Torisu K., Lee I.H., Liu J., Malide D., Combs C.A., Wu X.S., Rovira I.I., Fergusson M.M., Weigert R. (2013). Autophagy regulates endothelial cell processing, maturation and secretion of von Willebrand factor. Nat. Med..

[63] Trajkovic K., Hsu C., Chiantia S., Rajendran L., Wenzel D., Wieland F., Schwille P., Brügger B., Simons M. (2008). Ceramide triggers budding of exosome vesicles into multivesicular endosomes. Science.

[64] Hasanov Z., Ruckdeschel T., König C., Mogler C., Kapel S.S., Korn C., Spegg C., Eichwald V., Wieland M., Appak S. (2017). Endosialin promotes atherosclerosis through phenotypic remodeling of vascular smooth muscle cells. Arterioscler. Thromb. Vasc. Biol..

[65] Bennett M.R., Sinha S., Owens G.K. (2016). Vascular smooth muscle cells in atherosclerosis. Circ. Res..

[66] Chaulet H., Desgranges C., Renault M.A., Dupuch F., Ezan G., Peiretti F., Loirand G., Pacaud P., Gadeau A.P. (2001). Extracellular nucleotides induce arterial smooth muscle cell migration via osteopontin. Circ. Res..

[67] Pi S., Mao L., Chen J., Shi H., Liu Y., Guo X., Li Y., Zhou L., He H., Yu C. (2021). The P2RY12 receptor promotes VSMC-derived foam cell formation by inhibiting autophagy in advanced atherosclerosis. Autophagy.

[68] Robichaud S., Rasheed A., Pietrangelo A., Doyoung Kim A., Boucher D.M., Emerton C., Vijithakumar V., Gharibeh L., Fairman G., Mak E. (2022). Autophagy is differentially regulated in leukocyte and nonleukocyte foam cells during atherosclerosis. Circ. Res..

[69] Grootaert M.O.J., Moulis M., Roth L., Martinet W., Vindis C., Bennett M.R., De Meyer G.R.Y. (2018). Vascular smooth muscle cell death, autophagy and senescence in atherosclerosis. Cardiovasc. Res..

[70] Childs B.G., Zhang C., Shuja F., Sturmlechner I., Trewartha S., Fierro Velasco R., Baker D., Li H., van Deursen J.M. (2021). Senescent cells suppress innate smooth muscle cell repair functions in atherosclerosis. Nat. Aging..

[71] Liu Y.X., Yuan P.Z., Wu J.H., Hu B. (2021). Lipid accumulation and novel insight into vascular smooth muscle cells in atherosclerosis. J. Mol. Med..

[72] Forteza M.J., Ketelhuth D.F.J. (2022). Metabolism in atherosclerotic plaques: Immunoregulatory mechanisms in the arterial wall. Clin. Sci..

[73] Trus E., Basta S., Gee K. (2020). Who’s in charge here? Macrophage colony stimulating factor and granulocyte macrophage colony stimulating factor: Competing factors in macrophage polarization. Cytokine.

[74] Sottero B., Testa G., Gamba P., Staurenghi E., Giannelli S., Leonarduzzi G. (2022). Macrophage polarization by potential nutraceutical compounds: A strategic approach to counteract inflammation in atherosclerosis. Free. Radic. Biol. Med..

[75] Mushenkova N.V., Nikiforov N.G., Melnichenko A.A., Kalmykov V., Shakhpazyan N.K., Orekhova V.A., Orekhov A.N. (2022). Functional phenotypes of intraplaque macrophages and their distinct roles in atherosclerosis development and atheroinflammation. Biomedicines.

[76] Zhang Y., Choksi S., Chen K., Pobezinskaya Y., Linnoila I., Liu Z.G. (2013). ROS play a critical role in the differentiation of alternatively activated macrophages and the occurrence of tumor-associated macrophages. Cell Res..

[77] Madamanchi N.R., Vendrov A., Runge M.S. (2005). Oxidative stress and vascular disease. Arterioscler. Thromb. Vasc. Biol..

[78] Wang Y., Wang G.Z., Rabinovitch P.S., Tabas I. (2014). Macrophage mitochondrial oxidative stress promotes atherosclerosis and nuclear factor-kappaB-mediated inflammation in macrophages. Circ. Res..

[79] Tumurkhuu G., Shimada K., Dagvadorj J., Crother T.R., Zhang W., Luthringer D., Gottlieb R.A., Chen S., Arditi M. (2016). Ogg1-dependent DNA repair regulates NLRP3 inflammasome and prevents atherosclerosis. Circ. Res..

[80] Mohammadi A., Blesso C.N., Barreto G.E., Banach M., Majeed M., Sahebkar A. (2019). Macrophage plasticity, polarization and function in response to curcumin, a diet-derived polyphenol, as an immunomodulatory agent. J. Nutr. Biochem..

[81] Li B., Xia Y., Hu B. (2020). Infection and atherosclerosis: TLR-dependent pathways. Cell Mol. Life. Sci..

[82] Razani B., Feng C., Coleman T., Emanuel R., Wen H., Hwang S., Ting J.P., Virgin H.W., Kastan M.B., Semenkovich C.F. (2012). Autophagy links inflammasomes to atherosclerotic progression. Cell Metab..

[83] Jian X., Liu Y., Zhao Z., Zhao L., Wang D., Liu Q. (2019). The role of traditional Chinese medicine in the treatment of atherosclerosis through the regulation of macrophage activity. Biomed. Pharmacother..

[84] Kojima Y., Downing K., Kundu R., Miller C., Dewey F., Lancero H., Raaz U., Perisic L., Hedin U., Schadt E. (2014). Cyclin-dependent kinase inhibitor 2B regulates efferocytosis and atherosclerosis. J. Clin. Investig..

[85] Tabas I. (2010). Macrophage death and defective inflammation resolution in atherosclerosis. Nat. Rev. Immunol..

[86] Lewis G.F., Rader D.J. (2005). New insights into the regulation of HDL metabolism and reverse cholesterol transport. Circ. Res..

[87] Nissen S.E., Tsunoda T., Tuzcu E.M., Schoenhagen P., Cooper C.J., Yasin M., Eaton G.M., Lauer M.A., Sheldon W.S., Grines C.L. (2003). Effect of recombinant ApoA-I Milano on coronary atherosclerosis in patients with acute coronary syndromes: A randomized controlled trial. JAMA.

[88] Silverstein R.L., Febbraio M. (2009). CD36, a scavenger receptor involved in immunity, metabolism, angiogenesis, and behavior. Sci. Signal..

[89] Mallat Z., Hugel B., Ohan J., Lesèche G., Freyssinet J.M., Tedgui A. (1999). Shed membrane microparticles with procoagulant potential in human atherosclerotic plaques: A role for apoptosis in plaque thrombogenicity. Circulation.

[90] Hu Y., Zhang Z., Torsney E., Afzal A.R., Davison F., Metzler B., Xu Q. (2004). Abundant progenitor cells in the adventitia contribute to atherosclerosis of vein grafts in ApoE-deficient mice. J. Clin. Investig..

[91] Passman J.N., Dong X.R., Wu S.P., Maguire C.T., Hogan K.A., Bautch V.L., Majesky M.W. (2008). A sonic hedgehog signaling domain in the arterial adventitia supports resident Sca1+ smooth muscle progenitor cells. Proc. Natl. Acad. Sci. USA.

[92] Sun G., Gerecht S. (2009). Vascular regeneration: Engineering the stem cell microenvironment. Regen. Med..

[93] Xiao Q., Wang G., Luo Z., Xu Q. (2010). The mechanism of stem cell differentiation into smooth muscle cells. Thromb. Haemost..

[94] Xiao Q., Zeng L., Zhang Z., Hu Y., Xu Q. (2007). Stem cell-derived Sca-1+ progenitors differentiate into smooth muscle cells, which is mediated by collagen IV-integrin alpha1/beta1/alphav and PDGF receptor pathways. Am. J. Physiol. Cell Physiol..

[95] Xiao Q., Luo Z., Pepe A.E., Margariti A., Zeng L., Xu Q. (2009). Embryonic stem cell differentiation into smooth muscle cells is mediated by Nox4-produced H_2_O_2_. Am. J. Physiol. Cell Physiol..

[96] Pepe A.E., Xiao Q., Zampetaki A., Zhang Z., Kobayashi A., Hu Y., Xu Q. (2010). Crucial role of nrf3 in smooth muscle cell differentiation from stem cells. Circ. Res..

[97] Sharma A.K., Salmon M.D., Lu G., Su G., Pope N.H., Smith J.R., Weiss M.L., Upchurch G.R. (2016). Mesenchymal stem cells attenuate NADPH oxidase-dependent high mobility group box 1 production and inhibit abdominal aortic aneurysms. Arterioscler. Thromb. Vasc. Biol..

[98] Oh J., Lee Y.D., Wagers A.J. (2014). Stem cell aging: Mechanisms, regulators and therapeutic opportunities. Nat. Med..

[99] Wang C., Haas M., Yeo S.K., Sebti S., Fernández Á.F., Zou Z., Levine B., Guan J.L. (2022). Enhanced autophagy in Becn1(F121A/F121A) knockin mice counteracts aging-related neural stem cell exhaustion and dysfunction. Autophagy.

[100] Leeman D.S., Hebestreit K., Ruetz T., Webb A.E., McKay A., Pollina E.A., Dulken B.W., Zhao X., Yeo R.W., Ho T.T. (2018). Lysosome activation clears aggregates and enhances quiescent neural stem cell activation during aging. Science.

[101] Bautch V.L. (2011). Stem cells and the vasculature. Nat. Med..

[102] Bonacina F., Zhang X., Manel N., Yvan-Charvet L., Razani B., Norata G.D. (2024). Lysosomes in the immunometabolic reprogramming of immune cells in atherosclerosis. Nat. Rev. Cardiol..

[103] Seijkens T., Hoeksema M.A., Beckers L., Smeets E., Meiler S., Levels J., Tjwa M., de Winther M.P., Lutgens E. (2014). Hypercholesterolemia-induced priming of hematopoietic stem and progenitor cells aggravates atherosclerosis. FASEB J..

[104] Hedrick C.C. (2015). Lymphocytes in atherosclerosis. Arterioscler. Thromb. Vasc. Biol..

[105] Razeghian-Jahromi I., Karimi Akhormeh A., Razmkhah M., Zibaeenezhad M.J. (2022). Immune system and atherosclerosis: Hostile or friendly relationship. Int. J. Immunopathol. Pharmacol..

[106] Campbell K.A., Lipinski M.J., Doran A.C., Skaflen M.D., Fuster V., McNamara C.A. (2012). Lymphocytes and the adventitial immune response in atherosclerosis. Circ. Res..

[107] Engelen S.E., Robinson A.J.B., Zurke Y.X., Monaco C. (2022). Therapeutic strategies targeting inflammation and immunity in atherosclerosis: How to proceed?. Nat. Rev. Cardiol..

[108] Pattarabanjird T., Li C., McNamara C. (2021). B cells in atherosclerosis: Mechanisms and potential clinical applications. JACC Basic. Transl. Sci..

[109] Vellasamy D.M., Lee S.J., Goh K.W., Goh B.H., Tang Y.Q., Ming L.C., Yap W.H. (2022). Targeting immune senescence in atherosclerosis. Int. J. Mol. Sci..

[110] Marques A.R.A., Ramos C., Machado-Oliveira G., Vieira O.V. (2021). Lysosome (dys)function in atherosclerosis-A big weight on the shoulders of a small organelle. Front. Cell Dev. Biol..

[111] Tan J.X., Finkel T. (2023). Lysosomes in senescence and aging. EMBO Rep..

[112] Yang J., Rong S.J., Zhou H.F., Yang C., Sun F., Li J.Y. (2023). Lysosomal control of dendritic cell function. J. Leukoc. Biol..

[113] Noda N.N. (2024). Structural view on autophagosome formation. FEBS Lett..

[114] Zheng H., Li G., Min J., Xu X., Huang W. (2023). Lysosome and related protein degradation technologies. Drug. Discov. Today.

[115] Yao R., Shen J. (2023). Chaperone-mediated autophagy: Molecular mechanisms, biological functions, and diseases. MedComm.

[116] Dumitru C.A., Zhang Y., Li X., Gulbins E. (2007). Ceramide: A novel player in reactive oxygen species-induced signaling?. Antioxid. Redox. Signal..

[117] Schneider J.L., Suh Y., Cuervo A.M. (2014). Deficient chaperone-mediated autophagy in liver leads to metabolic dysregulation. Cell Metab..

[118] Kaushik S., Cuervo A.M. (2015). Degradation of lipid droplet-associated proteins by chaperone-mediated autophagy facilitates lipolysis. Nat. Cell Biol..

[119] Oku M., Sakai Y. (2018). Three Distinct Types of Microautophagy Based on Membrane Dynamics and Molecular Machineries. Bioessays.

[120] Madrigal-Matute J., Cuervo A.M., Sluimer J.C. (2022). Chaperone-mediated autophagy protects against atherosclerosis. Autophagy.

[121] Iacano A.J., Lewis H., Hazen J.E., Andro H., Smith J.D., Gulshan K. (2019). Miltefosine increases macrophage cholesterol release and inhibits NLRP3-inflammasome assembly and IL-1beta release. Sci. Rep..

[122] Zhou R., Yazdi A.S., Menu P., Tschopp J. (2011). A role for mitochondria in NLRP3 inflammasome activation. Nature.

[123] Nakahira K., Haspel J.A., Rathinam V.A., Lee S.J., Dolinay T., Lam H.C., Englert J.A., Rabinovitch M., Cernadas M., Kim H.P. (2011). Autophagy proteins regulate innate immune responses by inhibiting the release of mitochondrial DNA mediated by the NALP3 inflammasome. Nat. Immunol..

[124] Diao Y. (2021). Clematichinenoside AR alleviates foam cell formation and the inflammatory response in Ox-LDL-induced RAW264.7 cells by activating autophagy. Inflammation.

[125] Peng S., Xu L.W., Che X.Y., Xiao Q.Q., Pu J., Shao Q., He B. (2018). Atorvastatin inhibits inflammatory response, attenuates lipid deposition, and improves the stability of vulnerable atherosclerotic plaques by modulating autophagy. Front. Pharmacol..

[126] Epelman S., Liu P.P., Mann D.L. (2015). Role of innate and adaptive immune mechanisms in cardiac injury and repair. Nat. Rev. Immunol..

[127] Grebe A., Hoss F., Latz E. (2018). NLRP3 Inflammasome and the IL-1 Pathway in Atherosclerosis. Circ. Res..

[128] Duewell P., Kono H., Rayner K.J., Sirois C.M., Vladimer G., Bauernfeind F.G., Abela G.S., Franchi L., Nuñez G., Schnurr M. (2010). NLRP3 inflammasomes are required for atherogenesis and activated by cholesterol crystals. Nature.

[129] Rhoads J.P., Lukens J.R., Wilhelm A.J., Moore J.L., Mendez-Fernandez Y., Kanneganti T.D., Major A.S. (2017). Oxidized low-density lipoprotein immune complex priming of the Nlrp3 inflammasome involves TLR and FcgammaR cooperation and is dependent on CARD9. J. Immunol..

[130] Sheedy F.J., Grebe A., Rayner K.J., Kalantari P., Ramkhelawon B., Carpenter S.B., Becker C.E., Ediriweera H.N., Mullick A.E., Golenbock D.T. (2013). CD36 coordinates NLRP3 inflammasome activation by facilitating intracellular nucleation of soluble ligands into particulate ligands in sterile inflammation. Nat. Immunol..

[131] van Bruggen R., Köker M.Y., Jansen M., van Houdt M., Roos D., Kuijpers T.W., van den Berg T.K. (2010). Human NLRP3 inflammasome activation is Nox1-4 independent. Blood.

[132] Haslund-Vinding J., McBean G., Jaquet V., Vilhardt F. (2017). NADPH oxidases in oxidant production by microglia: Activating receptors, pharmacology and association with disease. Br. J. Pharmacol..

[133] Zheng F., Xing S., Gong Z., Xing Q. (2013). NLRP3 inflammasomes show high expression in aorta of patients with atherosclerosis. Heart Lung Circ..

[134] Paramel Varghese G., Folkersen L., Strawbridge R.J., Halvorsen B., Yndestad A., Ranheim T., Krohg-Sørensen K., Skjelland M., Espevik T., Aukrust P. (2016). NLRP3 inflammasome expression and activation in human atherosclerosis. J. Am. Heart. Assoc..

[135] Usui F., Shirasuna K., Kimura H., Tatsumi K., Kawashima A., Karasawa T., Hida S., Sagara J., Taniguchi S., Takahashi M. (2012). Critical role of caspase-1 in vascular inflammation and development of atherosclerosis in Western diet-fed apolipoprotein E-deficient mice. Biochem. Biophys. Res. Commun..

[136] Zhuang Y., Yasinta M., Hu C., Zhao M., Ding G., Bai M., Yang L., Ni J., Wang R., Jia Z. (2015). Mitochondrial dysfunction confers albumin-induced NLRP3 inflammasome activation and renal tubular injury. Am. J. Physiol. Renal. Physiol..

[137] Karasawa T., Takahashi M. (2017). Role of NLRP3 inflammasomes in atherosclerosis. J. Atheroscler. Thromb..

[138] Lao K.H., Zeng L., Xu Q. (2015). Endothelial and smooth muscle cell transformation in atherosclerosis. Curr. Opin. Lipidol..

[139] Hutchins P.M., Heinecke J.W. (2015). Cholesterol efflux capacity, macrophage reverse cholesterol transport and cardioprotective HDL. Curr. Opin. Lipidol..

[140] Nayer A., Ortega L.M. (2014). Catastrophic antiphospholipid syndrome: A clinical review. J. Nephropathol..

[141] Kume N., Cybulsky M.I., Gimbrone M.A. (1992). Lysophosphatidylcholine, a component of atherogenic lipoproteins, induces mononuclear leukocyte adhesion molecules in cultured human and rabbit arterial endothelial cells. J. Clin. Investig..

[142] Blankenberg S., Barbaux S., Tiret L. (2003). Adhesion molecules and atherosclerosis. Atherosclerosis.

[143] Burke-Gaffney A., Brooks A.V., Bogle R.G. (2002). Regulation of chemokine expression in atherosclerosis. Vascul. Pharmacol..

[144] Erl W., Weber P.C., Weber C. (1998). Monocytic cell adhesion to endothelial cells stimulated by oxidized low density lipoprotein is mediated by distinct endothelial ligands. Atherosclerosis.

[145] Rafieian-Kopaei M., Baradaran A. (2013). Combination of metformin with other antioxidants may increase its renoprotective efficacy. J. Renal. Inj. Prev..

[146] Steinbrecher U.P., Parthasarathy S., Leake D.S., Witztum J.L., Steinberg D. (1984). Modification of low density lipoprotein by endothelial cells involves lipid peroxidation and degradation of low density lipoprotein phospholipids. Proc. Natl. Acad. Sci. USA.

[147] Corsini A., Bernini F., Quarato P., Donetti E., Bellosta S., Fumagalli R., Paoletti R., Soma V.M. (1996). Non-lipid-related effects of 3-hydroxy-3-methylglutaryl coenzyme A reductase inhibitors. Cardiology.

[148] Fazio S., Major A.S., Swift L.L., Gleaves L.A., Accad M., Linton M.F., Farese R.V. (2001). Increased atherosclerosis in LDL receptor-null mice lacking ACAT1 in macrophages. J. Clin. Investig..

[149] Ghosh S. (2012). Early steps in reverse cholesterol transport: Cholesteryl ester hydrolase and other hydrolases. Curr. Opin. Endocrinol. Diabetes. Obes..

[150] Yeaman S.J. (2004). Hormone-sensitive lipase--new roles for an old enzyme. Biochem. J..

[151] Tabas I., Bornfeldt K.E. (2016). Macrophage phenotype and function in different stages of atherosclerosis. Circ. Res..

[152] Colin S., Chinetti-Gbaguidi G., Staels B. (2014). Macrophage phenotypes in atherosclerosis. Immunol. Rev..

[153] Nakashima Y., Wight T.N., Sueishi K. (2008). Early atherosclerosis in humans: Role of diffuse intimal thickening and extracellular matrix proteoglycans. Cardiovasc. Res..

[154] Tabas I., Williams K.J., Borén J. (2007). Subendothelial lipoprotein retention as the initiating process in atherosclerosis: Update and therapeutic implications. Circulation.

[155] Ross R. (1999). Atherosclerosis--an inflammatory disease. N. Engl. J. Med..

[156] Zhou X., Hansson G.K. (1999). Detection of B cells and proinflammatory cytokines in atherosclerotic plaques of hypercholesterolaemic apolipoprotein E knockout mice. Scand. J. Immunol..

[157] Chellan B., Rojas E., Zhang C., Hofmann Bowman M.A. (2018). Enzyme-modified non-oxidized LDL (ELDL) induces human coronary artery smooth muscle cell transformation to a migratory and osteoblast-like phenotype. Sci. Rep..

[158] Vengrenyuk Y., Nishi H., Long X., Ouimet M., Savji N., Martinez F.O., Cassella C.P., Moore K.J., Ramsey S.A., Miano J.M. (2015). Cholesterol loading reprograms the microRNA-143/145-myocardin axis to convert aortic smooth muscle cells to a dysfunctional macrophage-like phenotype. Arterioscler. Thromb. Vasc. Biol..

[159] Libby P., Buring J.E., Badimon L., Hansson G.K., Deanfield J., Bittencourt M.S., Tokgözoğlu L., Lewis E.F. (2019). Atherosclerosis. Nat. Rev. Dis. Primers..

[160] Durham A.L., Speer M.Y., Scatena M., Giachelli C.M., Shanahan C.M. (2018). Role of smooth muscle cells in vascular calcification: Implications in atherosclerosis and arterial stiffness. Cardiovasc. Res..

[161] Ceneri N., Zhao L., Young B.D., Healy A., Coskun S., Vasavada H., Yarovinsky T.O., Ike K., Pardi R., Qin L. (2017). Rac2 modulates atherosclerotic calcification by regulating macrophage interleukin-1beta production. Arterioscler. Thromb. Vasc. Biol..

[162] Zhu Y., McFarlane H.E. (2022). Regulation of cellulose synthesis via exocytosis and endocytosis. Curr. Opin. Plant. Biol..

[163] Mironov A.A., Mironov A., Sanavio B., Krol S., Beznoussenko G.V. (2023). Intracellular membrane transport in vascular endothelial cells. Int. J. Mol. Sci..

[164] Szewczyk-Roszczenko O.K., Roszczenko P., Shmakova A., Finiuk N., Holota S., Lesyk R., Bielawska A., Vassetzky Y., Bielawski K. (2023). The chemical inhibitors of endocytosis: From mechanisms to potential clinical applications. Cells.

[165] Wilfling F., Kaksonen M., Stachowiak J. (2023). Protein condensates as flexible platforms for membrane traffic. Curr. Opin. Cell Biol..

[166] Nagata S., Hanayama R., Kawane K. (2010). Autoimmunity and the clearance of dead cells. Cell.

[167] Liao X., Sluimer J.C., Wang Y., Subramanian M., Brown K., Pattison J.S., Robbins J., Martinez J., Tabas I. (2012). Macrophage autophagy plays a protective role in advanced atherosclerosis. Cell Metab..

[168] Berg R.D., Levitte S., O’Sullivan M.P., O’Leary S.M., Cambier C.J., Cameron J., Takaki K.K., Moens C.B., Tobin D.M., Keane J. (2016). Lysosomal disorders drive susceptibility to tuberculosis by compromising macrophage migration. Cell.

[169] Poon I.K., Lucas C.D., Rossi A.G., Ravichandran K.S. (2014). Apoptotic cell clearance: Basic biology and therapeutic potential. Nat. Rev. Immunol..

[170] Boada-Romero E., Martinez J., Heckmann B.L., Green D.R. (2020). The clearance of dead cells by efferocytosis. Nat. Rev. Mol. Cell Biol..

[171] Chang H.F., Schirra C., Pattu V., Krause E., Becherer U. (2023). Lytic granule exocytosis at immune synapses: Lessons from neuronal synapses. Front. Immunol..

[172] Buratta S., Tancini B., Sagini K., Delo F., Chiaradia E., Urbanelli L., Emiliani C. (2020). Lysosomal exocytosis, exosome release and secretory autophagy: The autophagic- and endo-lysosomal systems go extracellular. Int. J. Mol. Sci..

[173] Gibson M.S., Domingues N., Vieira O.V. (2018). Lipid and non-lipid factors affecting macrophage dysfunction and inflammation in atherosclerosis. Front. Physiol..

[174] Cheng X.W., Huang Z., Kuzuya M., Okumura K., Murohara T. (2011). Cysteine protease cathepsins in atherosclerosis-based vascular disease and its complications. Hypertension.

[175] Zhao C.F., Herrington D.M. (2016). The function of cathepsins B, D, and X in atherosclerosis. Am. J. Cardiovasc. Dis..

[176] de Nooijer R., Bot I., von der Thüsen J.H., Leeuwenburgh M.A., Overkleeft H.S., Kraaijeveld A.O., Dorland R., van Santbrink P.J., van Heiningen S.H., Westra M.M. (2009). Leukocyte cathepsin S is a potent regulator of both cell and matrix turnover in advanced atherosclerosis. Arterioscler. Thromb. Vasc. Biol..

[177] Libby P. (2006). Atherosclerosis: Disease biology affecting the coronary vasculature. Am. J. Cardiol..

[178] Xu K., Yang Y., Yan M., Zhan J., Fu X., Zheng X. (2010). Autophagy plays a protective role in free cholesterol overload-induced death of smooth muscle cells. J. Lipid. Res..

[179] Ouimet M., Franklin V., Mak E., Liao X., Tabas I., Marcel Y.L. (2011). Autophagy regulates cholesterol efflux from macrophage foam cells via lysosomal acid lipase. Cell Metab..

[180] Wild P.S., Zeller T., Schillert A., Szymczak S., Sinning C.R., Deiseroth A., Schnabel R.B., Lubos E., Keller T., Eleftheriadis M.S. (2011). A genome-wide association study identifies LIPA as a susceptibility gene for coronary artery disease. Circ. Cardiovasc. Genet..

[181] Proudfoot D., Skepper J.N., Hegyi L., Bennett M.R., Shanahan C.M., Weissberg P.L. (2000). Apoptosis regulates human vascular calcification in vitro: Evidence for initiation of vascular calcification by apoptotic bodies. Circ. Res..

[182] Mizushima N., Komatsu M. (2011). Autophagy: Renovation of cells and tissues. Cell.

[183] Henderson J.M., Weber C., Santovito D. (2021). Beyond self-recycling: Cell-specific role of autophagy in atherosclerosis. Cells.

[184] Tanaka M., Machida Y., Niu S., Ikeda T., Jana N.R., Doi H., Kurosawa M., Nekooki M., Nukina N. (2004). Trehalose alleviates polyglutamine-mediated pathology in a mouse model of Huntington disease. Nat. Med..

[185] Castillo K., Nassif M., Valenzuela V., Rojas F., Matus S., Mercado G., Court F.A., van Zundert B., Hetz C. (2013). Trehalose delays the progression of amyotrophic lateral sclerosis by enhancing autophagy in motoneurons. Autophagy.

[186] Kim J., Cheon H., Jeong Y.T., Quan W., Kim K.H., Cho J.M., Lim Y.M., Oh S.H., Jin S.M., Kim J.H. (2014). Amyloidogenic peptide oligomer accumulation in autophagy-deficient beta cells induces diabetes. J. Clin. Investig..

[187] Emanuel R., Sergin I., Bhattacharya S., Turner J., Epelman S., Settembre C., Diwan A., Ballabio A., Razani B. (2014). Induction of lysosomal biogenesis in atherosclerotic macrophages can rescue lipid-induced lysosomal dysfunction and downstream sequelae. Arterioscler. Thromb. Vasc. Biol..

[188] Viaud M., Ivanov S., Vujic N., Duta-Mare M., Aira L.E., Barouillet T., Garcia E., Orange F., Dugail I., Hainault I. (2018). Lysosomal cholesterol hydrolysis couples efferocytosis to anti-inflammatory oxysterol production. Circ. Res..

[189] Du H., Schiavi S., Wan N., Levine M., Witte D.P., Grabowski G.A. (2004). Reduction of atherosclerotic plaques by lysosomal acid lipase supplementation. Arterioscler. Thromb. Vasc. Biol..

[190] Dubland J.A., Francis G.A. (2015). Lysosomal acid lipase: At the crossroads of normal and atherogenic cholesterol metabolism. Front. Cell Dev. Biol..

[191] Marques A.R.A., Saftig P. (2019). Lysosomal storage disorders—Challenges, concepts and avenues for therapy: Beyond rare diseases. J. Cell Sci..

[192] Garner B., Priestman D.A., Stocker R., Harvey D.J., Butters T.D., Platt F.M. (2002). Increased glycosphingolipid levels in serum and aortae of apolipoprotein E gene knockout mice. J. Lipid. Res..

[193] Chatterjee S., Bedja D., Mishra S., Amuzie C., Avolio A., Kass D.A., Berkowitz D., Renehan M. (2014). Inhibition of glycosphingolipid synthesis ameliorates atherosclerosis and arterial stiffness in apolipoprotein E-/- mice and rabbits fed a high-fat and -cholesterol diet. Circulation.

[194] Liu C.L., Guo J., Zhang X., Sukhova G.K., Libby P., Shi G.P. (2018). Cysteine protease cathepsins in cardiovascular disease: From basic research to clinical trials. Nat. Rev. Cardiol..

[195] Sukhova G.K., Zhang Y., Pan J.H., Wada Y., Yamamoto T., Naito M., Kodama T., Tsimikas S., Witztum J.L., Lu M.L. (2003). Deficiency of cathepsin S reduces atherosclerosis in LDL receptor-deficient mice. J. Clin. Investig..

[196] Lutgens E., Lutgens S.P., Faber B.C., Heeneman S., Gijbels M.M., de Winther M.P., Frederik P., van der Made I., Daugherty A., Sijbers A.M. (2006). Disruption of the cathepsin K gene reduces atherosclerosis progression and induces plaque fibrosis but accelerates macrophage foam cell formation. Circulation.

[197] Kitamoto S., Sukhova G.K., Sun J., Yang M., Libby P., Love V., Duramad P., Sun C., Zhang Y., Yang X. (2007). Cathepsin L deficiency reduces diet-induced atherosclerosis in low-density lipoprotein receptor-knockout mice. Circulation.

[198] Kurdi A., De Doncker M., Leloup A., Neels H., Timmermans J.P., Lemmens K., Apers S., De Meyer G.R.Y., Martinet W. (2016). Continuous administration of the mTORC1 inhibitor everolimus induces tolerance and decreases autophagy in mice. Br. J. Pharmacol..

[199] Zhang K.S., Schecker J., Krull A., Riechert E., Jürgensen L., Kamuf-Schenk V., Burghaus J., Kiper L., Cao Ho T., Wöltje K. (2019). PRAS40 suppresses atherogenesis through inhibition of mTORC1-dependent pro-inflammatory signaling in endothelial cells. Sci. Rep..

[200] Tanaka Y., Guhde G., Suter A., Eskelinen E.L., Hartmann D., Lüllmann-Rauch R., Janssen P.M., Blanz J., von Figura K., Saftig P. (2000). Accumulation of autophagic vacuoles and cardiomyopathy in LAMP-2-deficient mice. Nature.

[201] Sergin I., Evans T.D., Zhang X., Bhattacharya S., Stokes C.J., Song E., Ali S., Dehestani B., Holloway K.B., Micevych P.S. (2017). Exploiting macrophage autophagy-lysosomal biogenesis as a therapy for atherosclerosis. Nat. Commun..

[202] Martina J.A., Diab H.I., Lishu L., Jeong-A L., Patange S., Raben N., Puertollano R. (2014). The nutrient-responsive transcription factor TFE3 promotes autophagy, lysosomal biogenesis, and clearance of cellular debris. Sci. Signal..

[203] Wang Y.T., Chen J., Li X., Umetani M., Chen Y., Li P.L., Zhang Y. (2019). Contribution of transcription factor EB to adipoRon-induced inhibition of arterial smooth muscle cell proliferation and migration. Am. J. Physiol. Cell Physiol..

[204] Wang Y.T., Li X., Chen J., McConnell B.K., Chen L., Li P.L., Chen Y., Zhang Y. (2019). Activation of TFEB ameliorates dedifferentiation of arterial smooth muscle cells and neointima formation in mice with high-fat diet. Cell Death. Dis..

[205] Sergin I., Razani B. (2014). Self-eating in the plaque: What macrophage autophagy reveals about atherosclerosis. Trends. Endocrinol. Metab..

[206] Zimmer S., Grebe A., Bakke S.S., Bode N., Halvorsen B., Ulas T., Skjelland M., De Nardo D., Labzin L.I., Kerksiek A. (2016). Cyclodextrin promotes atherosclerosis regression via macrophage reprogramming. Sci. Transl. Med..

[207] Coisne C., Hallier-Vanuxeem D., Boucau M.C., Hachani J., Tilloy S., Bricout H., Monflier E., Wils D., Serpelloni M., Parissaux X. (2016). Beta-cyclodextrins decrease cholesterol release and ABC-associated transporter expression in smooth muscle cells and aortic endothelial cells. Front. Physiol..

